# Physiological and pharmacological stimulation for in vitro maturation of substrate metabolism in human induced pluripotent stem cell-derived cardiomyocytes

**DOI:** 10.1038/s41598-021-87186-y

**Published:** 2021-04-08

**Authors:** Colleen A. Lopez, Heba Hussain A. A. Al-Siddiqi, Ujang Purnama, Sonia Iftekhar, Arne A. N. Bruyneel, Matthew Kerr, Rabia Nazir, Maria da Luz Sousa Fialho, Sophia Malandraki-Miller, Rita Alonaizan, Fatemeh Kermani, Lisa C. Heather, Jan Czernuszka, Carolyn A. Carr

**Affiliations:** 1 Department of Physiology, Anatomy and Genetics, University of Oxford, Sherrington Building, Parks Road, Oxford, OX1 3PT UK; 2 Department of Material Science, University of Oxford, Parks Road, Oxford, OX1 3PH UK; 3 School of Medicine’s Cardiovascular Medicine Research Center, Yale University, 300 George Street, New Haven, CT 06511 USA; 4 Qatar Biomedical Research Institute, Hamad Bin Khalifa University, Education City, P.O. Box 34110, Doha, Qatar; 5 Interdisciplinary Research Centre in Biomedical Materials, COMSATS University Islamabad, Lahore Campus, 1.5 KM Defence Road Off Raiwind Road, Lahore, Pakistan

**Keywords:** Cell biology, Stem cells

## Abstract

Human induced pluripotent stem cell-derived cardiomyocytes (hiPSC-CMs) enable human cardiac cells to be studied in vitro, although they use glucose as their primary metabolic substrate and do not recapitulate the properties of adult cardiomyocytes. Here, we have explored the interplay between maturation by stimulation of fatty acid oxidation and by culture in 3D. We have investigated substrate metabolism in hiPSC-CMs grown as a monolayer and in 3D, in porous collagen-derived scaffolds and in engineered heart tissue (EHT), by measuring rates of glycolysis and glucose and fatty acid oxidation (FAO), and changes in gene expression and mitochondrial oxygen consumption. FAO was stimulated by activation of peroxisome proliferator-activated receptor alpha (PPARα), using oleate and the agonist WY-14643, which induced an increase in FAO in monolayer hiPSC-CMs. hiPSC-CMs grown in 3D on collagen-derived scaffolds showed reduced glycolysis and increased FAO compared with monolayer cells. Activation of PPARα further increased FAO in cells on collagen/elastin scaffolds but not collagen or collagen/chondroitin-4-sulphate scaffolds. In EHT, FAO was significantly higher than in monolayer cells or those on static scaffolds and could be further increased by culture with oleate and WY-14643. In conclusion, a more mature metabolic phenotype can be induced by culture in 3D and FAO can be incremented by pharmacological stimulation.

## Introduction

Cardiac metabolism and function are inherently linked, as substrate metabolism generates the energy which enables the heart to contract^1^. The metabolic characteristics of the human heart vary during development and in disease due to changes in substrate availability and energetic demand ^1^. With the development of protocols to differentiate human pluripotent stem cells into beating cardiomyocytes (CMs), human heart cells can now be studied in vitro^2^. This promises valuable insights into the biology of human CMs. However, it is hindered by the fact that human induced pluripotent stem cell-derived cardiomyocytes (hiPSC-CMs) have a very immature phenotype and do not fully recapitulate the properties of adult heart cells, even when cultured for prolonged periods^3^. This is, in part, due to the fact that hiPSC-CMs are frequently grown in 2D in high glucose media so that the cells have limited metabolic demand and are exposed to non-physiological concentrations of nutrients^4^.

Mammalian cells metabolize a variety of substrates, depending on nutrient availability, oxygen supply, and energy requirements for processes such as mechanical work, thermogenesis, ion transport, and synthesis of cell components^5^. The adult heart is required to maintain cardiac function under all circumstances, necessitating the production of around 4–6 kg of adenosine triphosphate (ATP) per day^1,6^. The healthy heart uses exogenous free fatty acids (FFA; ~ 70% of ATP production) and glucose (~ 20% of ATP production) via the oxidation of acetyl-Coenzyme A (acetyl-CoA) in the mitochondrial tricarboxylic acid (TCA) cycle^1,7^. In contrast, cells cultured in vitro grow in high glucose media, which provides all the ATP necessary, predominantly via glycolysis^4,8^. This resembles the metabolism in the foetal heart, which also has a highly glycolytic phenotype^9^. During embryonic development, there is a large mitochondrial biogenic drive to increase mitochondrial number and upregulate electron transport chain function. The cardiac mitochondrial networks restructure and the mitochondrial membrane potential increases, as TCA cycle metabolism is upregulated^10^.

Until recently, metabolic changes in the human heart could only be measured using non-invasive techniques such as magnetic resonance spectroscopy and positron emission tomography or deduced from studies in animal models. However, CMs from laboratory animals can be substantially different from human CMs^11^. The development of protocols to differentiate human embryonic stem cells or induced pluripotent stem cells (hiPSCs) into beating CMs means that we can now investigate characteristics of human heart cells in the lab, providing useful tools for drug development, toxicology and therapeutic applications^12^. Although the restructuring of the glycolytic network and upregulation of oxidative metabolism has been reported during differentiation of embryonic stem cells into CMs^13,14^, these differentiated cells remain predominantly glycolytic and do not develop a fully mature metabolic capability^15^. Beating CMs differentiated from pluripotent stem cells have immature calcium handling^16^ and a response to cardioactive drugs, such as isoprenaline, which is more akin to those of the embryonic or failing human heart^17^. Over time the differentiated cells begin to mature, but they require at least 6–8 weeks of culture before the expression profile of cardiac genes and myofibril alignment begin to resemble those of the adult heart^3,18^. A more mature phenotype can also be induced by treatment with molecules associated with changes in the developing heart, such as the thyroid hormone triiodothyronine^19^, or by using a glucose-free maturation medium containing alternative oxidizable substrates^20^. Most recently, Yang et al.have treated hiPSC-CMs grown in 2D with media containing both glucose and a cocktail of fatty acids designed to match levels found in newborn infants^21^, Ramachandra et al. treated cardiac clusters with media containing glucose and a 1:1 mixture of palmitate and oleate^22^ and Horikoshi et al. used a glucose-free maturation medium containing fatty acids^23^. Nevertheless, hiPSC-CMs that are grown as a monolayer are unlikely to fully recapitulate the metabolic phenotype of cardiac cells in vivo.

Tissue engineering has become an attractive avenue to induce a more mature phenotype in hiPSC-CMs by creating a culture condition that better mimics the environment of the cell in vivo^15^. Mills et al. have used beating cardiac organoids to optimize maturation by adjusting a range of parameters, including metabolic substrates, and found that a switch to fatty acid metabolism was a key factor in maturation^24^. Here, we have explored the role of both physiological and pharmacological stimulation in the maturation of substrate metabolism using the interplay between maturation by stimulation of fatty acid oxidation (FAO) and by culture in 3D. hiPSC-CMs were grown as a 2D monolayer, in 3D culture on collagen-derived scaffolds interlaced with extracellular-matrix materials or in engineered heart tissue (EHT). We investigated substrate metabolism in hiPSC-CMs grown as a monolayer and in 3D, with and without stimulation of fatty acid oxidation by activation of the peroxisome proliferator-activated receptor alpha (PPARα) using oleate (OA) and the agonist pirinixic acid (WY-14643)^25^. PPARα is a critical component in driving metabolic maturation in the heart post-birth by upregulating FAO (Fig. S1)^26,27^ and we have previously shown that addition of OA led to maturation of differentiating mouse cardiac progenitors^28^. Here we have found that a metabolic switch with decreased glycolysis and increased FAO can be induced by switching to 3D culture and requiring the cells to contract against a restraint as is seen with the EHT. We found that stimulation of PPARα further increased oxidative metabolism under some conditions, but not all.

## Results

### hiPSC-CMs mature over long-term culture

Human iPSCs were differentiated into beating CMs using a protocol modified from that of Lian et al.^29^, which produced 75–90% cells positive for alpha-actinin (Fig. S2). Oxygen consumption and the rate of glycolysis were measured in hiPSCs and beating hiPSC-CMs after 1 month of differentiation. When the cells were respiring on oleate, the rate of OCR was twofold higher in hiPSC-CMs than hiPSCs (Fig. S3a). The increased ability to respire on oleate was supported by an increase in mRNA expression for the fatty acid transporter *CD36*, the rate-controlling enzyme carnitine palmitoyl transferase 1B (*CPT1B*), *PPAR*α, and the long-chain acyl CoA synthase *ACSL1* (Fig. S3b). In contrast there was no significant difference in OCR between hiPSCs and hiPSC-CMs respiring on pyruvate and malate (Fig. S3c). However, the rate of glycolytic flux was significantly higher in hiPSC-CMs than in hiPSCs, suggesting an increase in energy generation from glycolysis but no increase in flux through to glucose oxidation (Fig. S3d). These changes were associated with an increased expression of the insulin responsive glucose transporter *GLUT4* and of 6-phosphofructokinase (*PFKM*) which catalyses the irreversible rate-limiting step in glycolysis (Fig. S3e). Expression of the pyruvate dehydrogenase alpha-subunit (*PDHA1)* and of pyruvate dehydrogenase kinase 4 (*PDK4),* which controls the flux of pyruvate into the TCA cycle were also increased. Interestingly, expression of oxoglutarate dehydrogenase (*OGDH),* that catalyzes the decarboxylation of α-ketoglutarate in the TCA cycle, decreased. Expression of peroxisome proliferator activated receptor gamma coactivator 1-alpha (*PGC1α*), an indicator of mitochondrial biogenesis, increased and was associated with an increase in mitochondrial number and mitochondrial membrane potential (Fig. S3f, g).

To investigate the time course of changes in gene expression during long-term maturation, mRNA expression was quantified in hiPSC-CMs, normalized to that early in differentiation at day 7, and from 1 to 3 months of differentiation, normalized to expression at 1 month (Fig. S4). The cardiac genes, myosin light chain 7 (*MYL7*) and myosin heavy chain 6 and 7 (*MYH6, MYH7*) were upregulated during the first weeks of differentiation and expression of *MYH7* continued to increase over 3 months of culture. Expression of the sarcoplasmic reticulum calcium transport ATPase (*SERCA2α*) increased in the first weeks of differentiation but did not change significantly thereafter, and there were no significant changes in expression of the calcium handling genes (calsequestrin 2 [*CASQ2*] or the ryanodine receptor [*RYR2*]) or of the gap junction protein connexin 43 (*CX43*). Expression of *GLUT4* and *PDK4* increased throughout time in culture. In contrast, that of citrate synthase (*CS*) and *PGC1α* did not increase significantly until 2–3 months. Thus, after an initial upregulation of oxidative metabolism, further maturation of hiPSC-CMs continued for at least 3 months.

### Fatty acid oxidation can be induced by activation of the PPARα pathway

**Figure 1:**
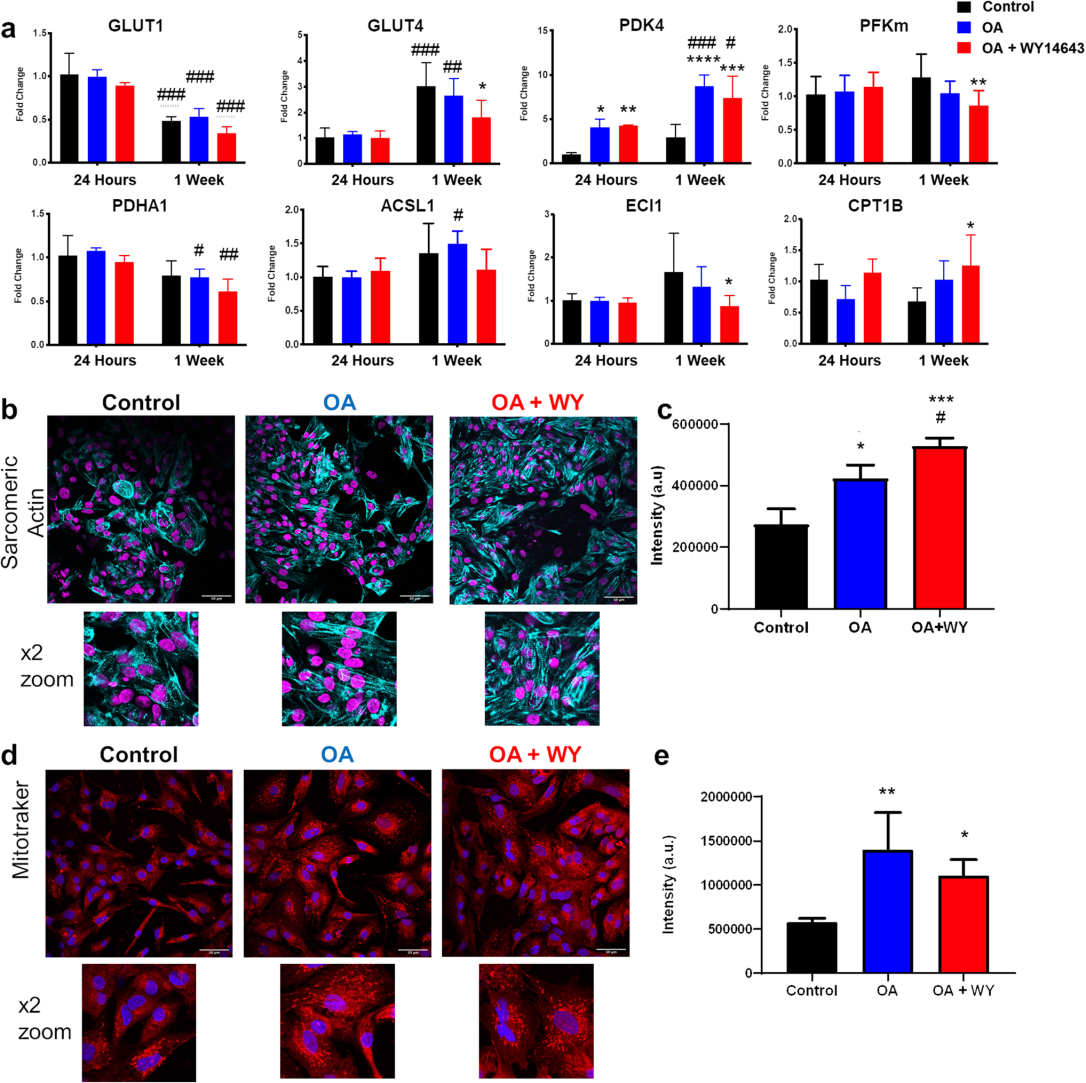
(**a**) Changes in mRNA gene expression of day 12 hiPSC-CMs seeded as a monolayer on RF-Matrigel and cultured for 24 h or 1 week with OA ± WY-14643; *,**,***,**** = *p* < 0.05, 0.01, 0.001, 0.0001 compared with control hiPSC-CMs cultured for 24 h; #,##,###,#### = *p* < 0.05, 0.01, 0.001, 0.0001 compared with control cells at the same time point (n = 4). Immunostaining for (b) α-Sarcomeric Actinin (cyan with DAPI in magenta) or (d) Mitotracker CMXRos (red with DAPI in blue) in day 12-15 hiPSC-CMs seeded as a monolayer on RF-Matrigel, as control or after treatment for 1 week with OA ± WY-14643; scale bar is 50 µM with × 2 zoom below; signal intensity is quantified in (**c**, **e**) *, **p < 0.05, 0.01 compared with control hiPSC-CMs (n = 4).

hiPSC-CMs do not need to upregulate pathways associated with FAO substantially whilst in culture in high glucose media containing little to no fatty acids. Therefore, fatty acid metabolism was stimulated after 15 days of differentiation by the addition of OA with or without the PPARα agonist WY-14643. Cells were treated with OA alone or with OA plus WY-14643 at concentrations of 60, 120, 180 and 240 μM for 24 h and 7 days and the cell number counted using a CCK-8 assay (Fig. S5a, b). Treatment with WY-14643 caused a dose-dependent decrease in cell number after 24 h and 7 days (*p* < 0.05 for both time points) with a significant decrease in cell number at the highest concentration. *PDK4* expression, indicative of PPARα activation, was measured after 24 h (Fig. S5c). Addition of both 120 and 180 μM significantly increased *PDK4* expression, with no difference in expression between the two concentrations. Protein levels of sarcomeric α-actinin were unchanged by treatment with doses up to 180 μM (Fig. S5d). A dose of 120 μM WY-14643 was therefore chosen for further experiments. Treatment with OA and WY-14643 for 8 h was sufficient to induce changes in metabolic genes with a decrease the expression of *GLUT4* and *PFKM* and an increase in the expression of *CPT1B* by twofold and of the acyl-CoA dehydrogenase, *ACADM* (Fig. S6). Similarly, expression of enoyl CoA isomerase (*ECI1*)*,* essential for the mitochondrial beta-oxidation of unsaturated fatty acids, and of *PDK4* increased over 8 h. hiPSC-CMs were then treated with OA and WY-14643 for 24 h and 1 week and changes in mRNA expression after 1 week were compared to control cells after 24 h of culture (Fig. 1a). There was a shift in expression of the glucose transporters from *GLUT1* to *GLUT4* over 1 week of culture, whilst treatment with OA + WY-14643 inhibited increased expression of *GLUT4*. Expression of *PDK4* increased with time and increased tenfold with treatment with OA, but there was no further increase after addition of WY-14643. Small changes were seen in the expression of *PDHA1*, which decreased, and of *ACSL1*, which increased after 1 week of treatment with OA. Expression of sarcomeric actinin was investigated using immunostaining and shown to increase after the addition of OA, with a further increase on addition of WY-14643 (Fig. 1b, c). This was in contrast to the data in the dose response experiment shown in Fig. S5, where we saw a non-significant increase in protein levels for *α-*sarcomeric actinin between cells treated with OA alone and those treated with OA and 120 µM WY-14643, measured using Western blotting. This difference may in part be due to a difference in number of samples (n = 4 for immunostaining and n = 3 for Western blotting) and in part because protein expression was normalized to total protein in the sample for Western blotting, whilst in the immunostaining experiment, signal intensity was normalized to cell number, Similarly, MitoTracker staining showed an increase in intensity after the 1-week treatment with OA ± WY-14643 (Fig. 1d, e). Mitotracker staining also suggested increased formation of mitochondrial networks after treatment with OA.

**Figure 2:**
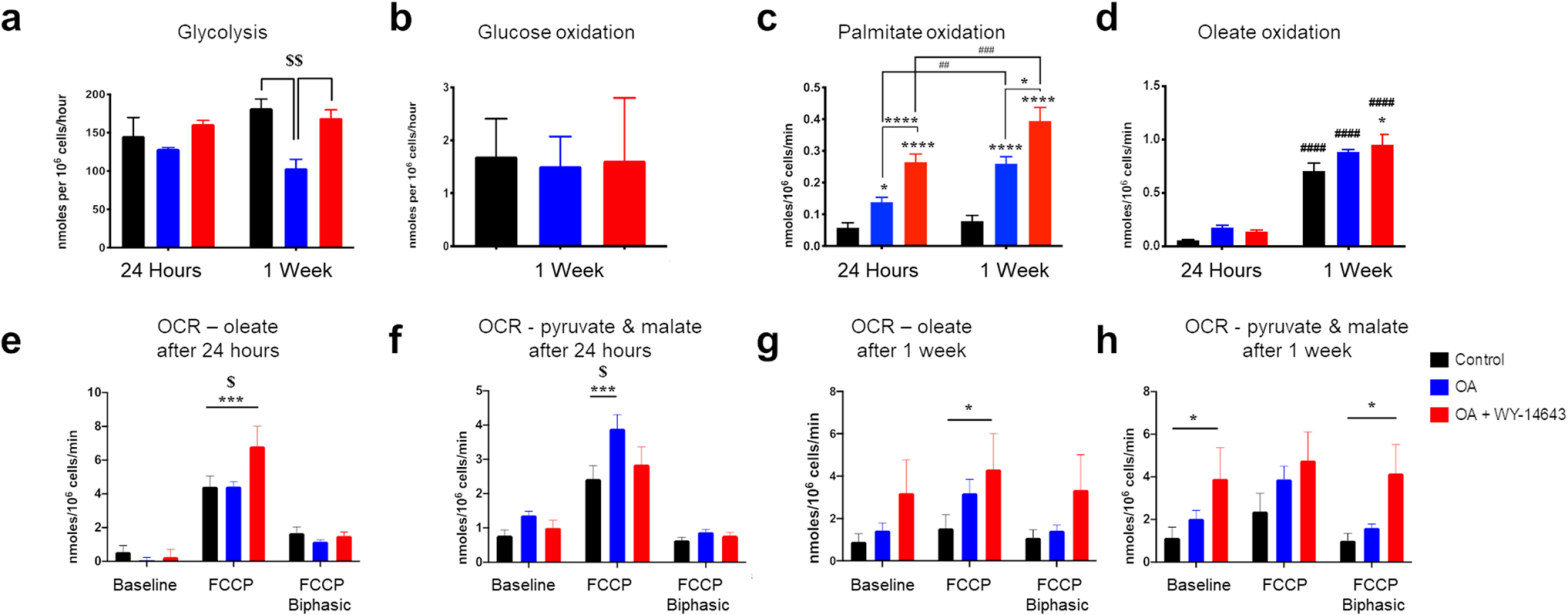
Rates of (**a**) glycolysis (n = 4), and of oxidation of (**b**) glucose (n = 8), (**c**) palmitate (n = 16) and (**d**) oleate (n = 4) were measured in day 12–15 hiPSC-CMs after culture for 24 h or 1 week with OA + / − WY-14643. Cellular oxygen consumption rate (OCR) was measured using the Clark-type oxygen electrode in hiPSC-CMs respiring on oleate (**e**, **g**) or pyruvate & malate (**f**, **h**); *,**,***,****p < 0.05, 0.01, 0.001, 0.0001 compared with control or treated hiPSC-CMs at the same time point as indicated; #, ##, ###, ####*p* < 0.05, 0.01, 0.001, 0.0001 compared with cells treated for 24 h; $, $$*p* < 0.05, 0.01 compared with hiPSC-CMs + OA (n = 6–14).

Rates of glycolysis did not change with time in culture but, after treatment for 1 week, were decreased by treatment with OA, but not by treatment with OA + WY-14643 (Fig. 2a). There was no change in glucose oxidation after treatment with OA ± WY-14643 for 1 week (Fig. 2b). Culture with OA for 24 h was sufficient to increase palmitate oxidation, with a further increase after addition of WY-14643 and after 1 week of treatment (Fig. 2c). Interestingly, culture with OA for 24 h induced a similar increase in oleate oxidation to that seen with palmitate, albeit not significant, but oleate oxidation increased in all cells to a much greater extent after 1 week in culture, with a further small increase in cells treated with OA + WY-14643 (Fig. 2d).

Cellular OCR increased in hiPSC-CMs respiring on OA after 24 h of treatment with OA + WY-14643 compared with control cells or those treated with OA alone (Fig. 2e). In contrast, oxygen consumption in hiPSC-CMs respiring on pyruvate and malate was highest in cells cultured with OA compared with control cells and those treated with OA + WY-14643 (Fig. 2f). After 1 week of culture with OA + WY-14643, there was an increase in OCR at baseline when respiring on pyruvate and malate, which did not increase further in response to the uncoupler FCCP, suggesting a more oxidative metabolism at baseline (Fig. 2g, h).

### 3D Culture of hiPSC-CMs increases cell maturation

**Figure 3:**
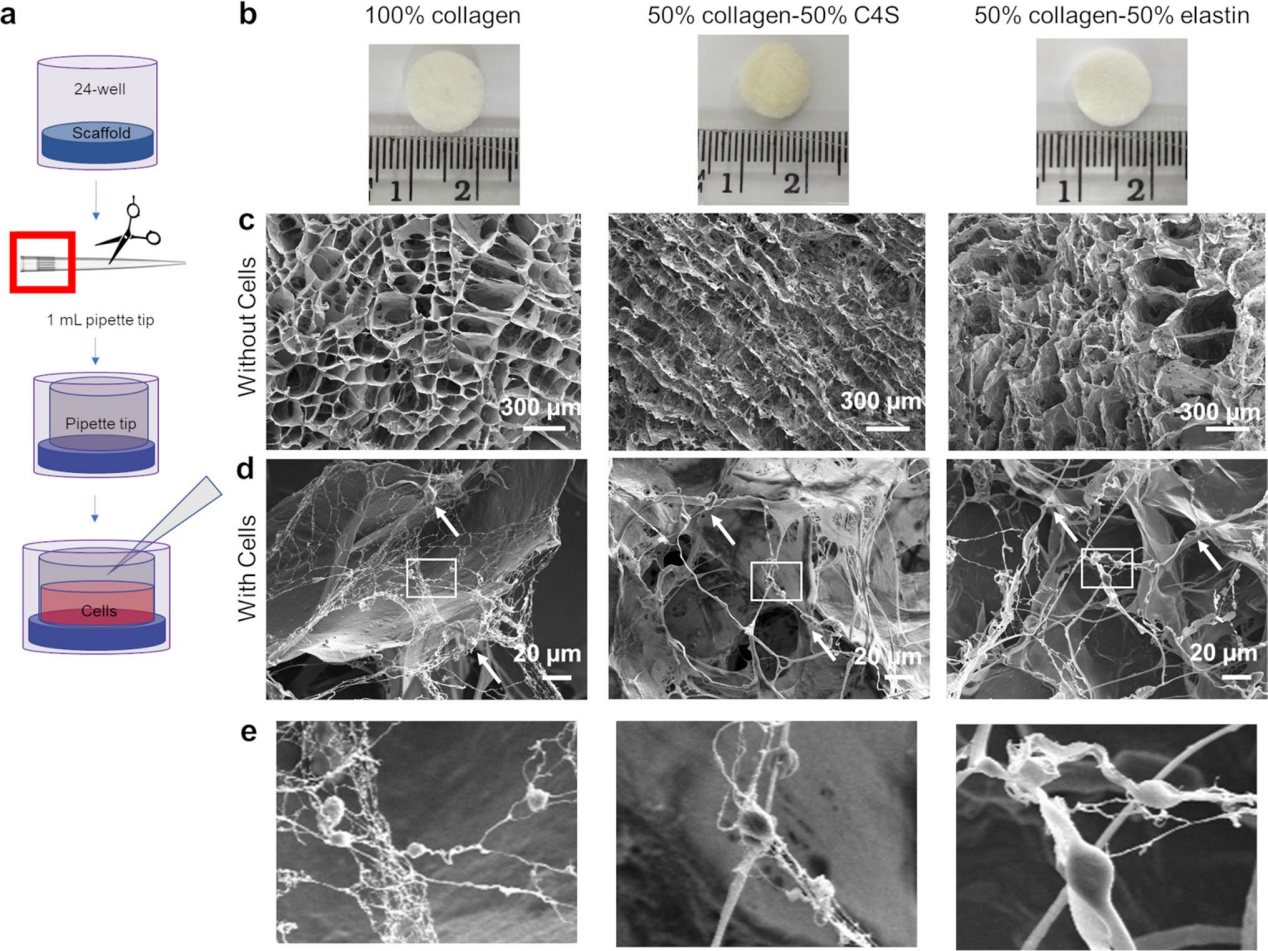
(**a**) Schematic of loading collagen-derived scaffolds with hiPSC-CMs; (**b**) photos of the top view of each of the different collagen-derived disc-shaped scaffolds next to a centimetre ruler; (**c**–**e**) scanning electron microscopy images of the scaffolds, (**c**) without cells in the top row (scale bar is 300 μm) and (**d**) with cells, indicated by white arrows in the bottom row (scale bar is 20 μm) and with × 5 zoom of boxed area shown in (**e**).

To determine whether culture in 3D encouraged hiPSC-CMs to adopt a more mature metabolic phenotype, cells were grown on porous scaffolds prepared from extracellular matrix proteins. Scaffolds were generated by freeze-drying solutions of collagen 1 alone or in combination with elastin or the glycosaminoglycan chondroitin-4-sulfate (C4S)^30,31^. The scaffolds were approximately 1.3 cm in diameter, designed to cover the bottom of a well of a 24-well plate, and the top of a 1 ml pipette tip was placed on the top of each scaffold to ensure that the majority of cells were retained within the scaffold on seeding (Fig. 3a, b). SEM images of each of the collagen-derived compositions showed that the pore sizes in the scaffold were around 100–300 µm (Fig. 3c). hiPSC-CMs appeared to have formed web-like networks for attachment to the host scaffold (Fig. 3d, e), which were not evident when cardiosphere-derived cells were cultured on similar scaffolds^31^.

**Figure 4:**
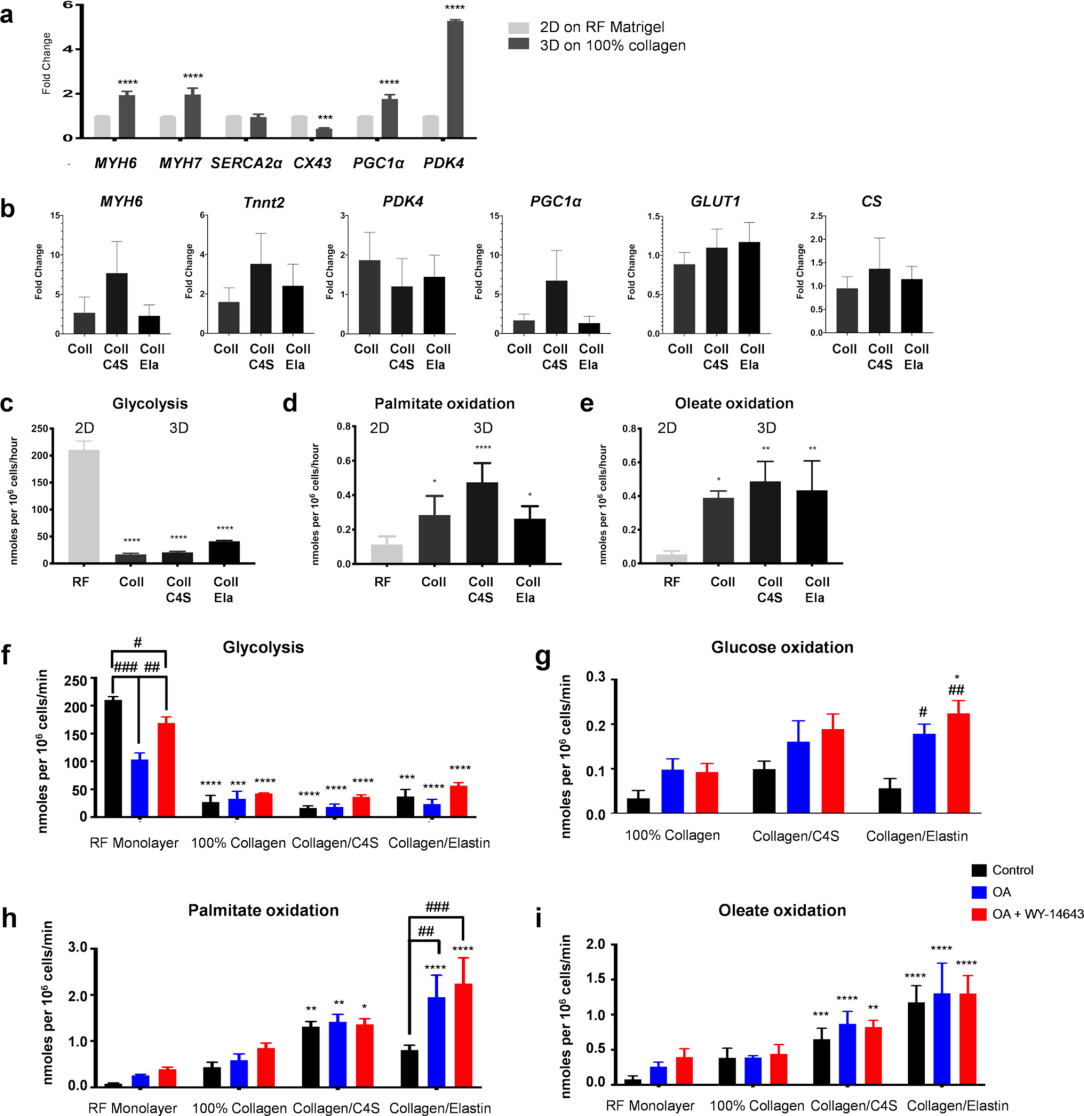
(**a**) Changes in mRNA expression of day 12–15 hiPSC-CMs seeded as a monolayer on RF-Matrigel (RF) or in 3D on scaffolds of 100% collagen 1 (100% Coll) and cultured for 1 week. (n = 3) (**b**) Changes in mRNA expression of day 12–30 hiPSC-CMs seeded on scaffolds of collagen (100% Coll), 1:1 collagen:chondroitin-4-sulphate (Coll/C4S) or 1:1 collagen:elastin (Coll/Ela) and cultured for 1 week (n = 3). Rates of (**c**) glycolysis, and oxidation of (**d**) palmitate and (**e**) oleate by day 12–15 hiPSC-CMs seeded as a monolayer or in 3D on collagen scaffolds and cultured for 1 week in control media. *, **, ***p < 0.5, 0.01, 0.001, respectively compared with hiPSC-CMs on RF-Matrigel. Rates of (f) glycolysis and oxidation of (**g**) glucose, (**h**) palmitate and (**i**) oleate by day 12–15 hiPSC-CMs seeded as a monolayer or in 3D and cultured for 1 week in control media or with addition of OA ± WY-14643. *, **, ***p < 0.5, 0.01, 0.001, respectively compared with hiPSC-CMs on RF-Matrigel; #, ##, ###, ####*p* < 0.5, 0.01, 0.001, 0.001, respectively compared to the control in their respective group (n = 6–12).

Changes in mRNA expression were measured in cells grown as monolayers on reduced factor (RF) Matrigel or in 3D on collagen scaffolds for 1 week. hiPSC-CMs grown in 3D on a scaffold of 100% collagen showed increased expression of *MYH6*, *MYH7*, *PGC1α,* and *PDK4* (Fig. 4a) but the scaffold composition did not significantly affect expression of the genes studied (Fig. 4b). Rates of glycolysis were significantly reduced in hiPSC-CMs grown in 3D, concomitant with an increase in the rates of FAO, despite the fact that the cells had been cultured in glucose only medium (Fig. 4c–e). Addition of OA ± WY-14643 did not change rates of glucose metabolism or FAO in hiPSC-CMs grown on scaffolds of collagen or collagen/C4S (Fig. 4f–i) but did increase glucose and palmitate oxidation in hiPSC-CMs grown on collagen/elastin scaffolds.

**Figure 5:**
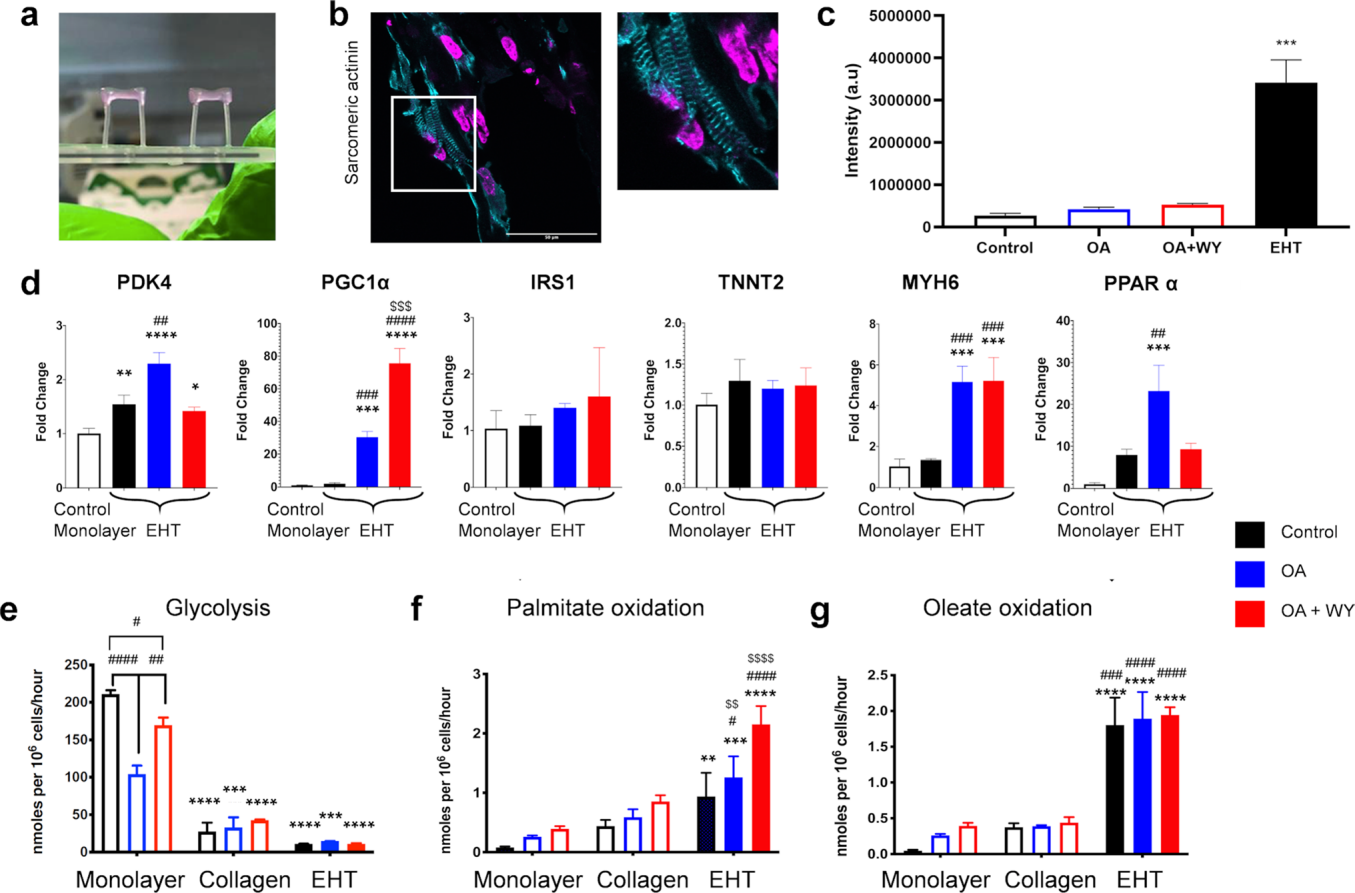
(**a**) EHT attached to the silicon posts; (**b**) immunostaining for α-Sarcomeric Actinin (Actinin-cyan, DAPI-magenta) in a section through an EHT, scale bar is 50 µm with × 2 zoom to the right; signal intensity is quantified in (**c**) compared with that for cells in 2D shown in Fig. 1b, indicated as open bars. (**d**) Changes in mRNA expression in day 15 hiPSC-CMs seeded as a monolayer on RF-Matrigel or in EHTs and treated with OA ± WY-14643 for 1 week. Rates of (**e**) glycolysis and oxidation of (**f**) palmitate and (**g**) oleate in EHT treated with OA ± WY-14643 for 1 week and compared with data for cells cultured in 2D or on 100% collagen scaffold, shown in Fig. 4, indicated as open bars. *, **, ***p < 0.5, 0.01, 0.001, respectively compared with hiPSC-CMs on RF-Matrigel; #, ##, ###, ####*p* < 0.5, 0.01, 0.001, 0.001, respectively compared to the control in their respective group; $$, $$$, $$$$*p* < 0.01, 0.001, 0.001 compared to OA-treated EHTs for (**d**) or  hiPSC-CMs on 100% collagen for (**e**–**g**). Data in (**c**–**d**) were compared using a one way ANOVA and in (**e**–**g**) were compared between all groups using a two-way ANOVA, with a Tukey post-hoc test in all cases. (n = 3 for EHTs).

In comparison to monolayers and scaffolds, cells in EHT have to contract against a restraining post (Fig. 5a) and are therefore doing more work, which has been shown to increase oxidative metabolism^32^. hiPSC-CMs grown in EHT showed higher sarcomeric actinin expression and clearer striations than cells cultured in 2D (Fig. 5b, c). Following treatment with OA ± WY-14643 for 1 week, changes in mRNA expression were investigated. Treatment with OA alone induced an increase in *PDK4*, *PGC1α*, *MYH6* and *PPAR-α* expression (Fig. 5d). There was an additional increase in *PGC1α* expression after treatment with OA + WY-14643, but levels of *PDK4* and *PPAR-α* were comparable to those in control EHT cells. EHT had a significantly increased rate of FAO (both palmitate and oleate oxidation) and a substantial decrease in glycolysis compared to that of cells in the RF-Matrigel-coated monolayer (Fig. 5e–g). The rate of palmitate oxidation was lower than that of oleate but was increased after treatment with OA + WY-14643 (Fig. 5f, g). Mitochondrial membrane potential and α-sarcomeric actinin were assessed in hiPSC-CMs in the EHT treated with OA ± WY-14643 but no overt changes in expression were detected (Figure S7).

## Discussion

hiPSC-CMs are immature and reflect more of a foetal phenotype than that of an adult CM^18,33–38^ despite a range of different strategies for maturation including long-term culture^39–41^, applied mechanical and electrical stimulation^42–44^, genetic approaches^45,46^, substrate stiffness^47^, addition of small compounds^19^ and 3D culture^24,32,48–50^. As shown by others^3^, we confirmed that hiPSC-CMs mature slowly over time with changes in structural genes occurring during the first month of differentiation and continuing to increase for up to 3 months. We therefore aimed to promote maturation of hiPSC-CMs through stimulating FAO by PPARα activation via the addition of the PPARα agonist WY-14643^51^ and of oleate into the culture media, in combination with a tissue engineering approach to grow cells in 3D.

It has previously been shown that PPARα is a critical component in driving metabolic maturation in the heart post-birth^26,27^. The expression of PPAR-α peaks twice during differentiation of mouse embryonic stem cells to CMs^52^ and treatment with PPARα agonists during differentiation increased the number of spontaneously contracting embryoid bodies, with an associated increase in expression of cardiac genes. It has been shown that switching to glucose-free medium forced hiPSC-CMs to shift to oxidative phosphorylation, which induced a phenotype more similar to human CMs than previously reported^20^. Similarly Rana et al.^53^ modified basal medium using combinations of high glucose or galactose, optionally with FAs, and found that hiPSC-CMs would only increase oxidative metabolism for their ATP production when glucose was eliminated from the medium. Interestingly, the ATP content of the hiPSC-CMs was not changed by the culture medium used, suggesting that the monolayer cells could generate sufficient ATP from glycolysis and only switched to oxidative metabolism when there was no alternative. More recently, addition of FAs to glucose-containing medium has been shown to induce structural maturation and enhanced oxidative metabolism and contractile function^21,24,54^.

Tissue engineering has become an attractive avenue used to induce a more mature phenotype in hiPSC-CMs by creating a more physiological relevant culture condition that better mimics the environment of the cell in vivo^15^. Bian et al.^55^ cultured neonatal rat CMs in a 3D environment and after 3 weeks saw aligned, electromechanically coupled CMs with capillary like structures, and improved calcium handling properties. Cardiac muscle strips fabricated from embryonic stem cell-derived cardiomyocytes and stromal cells in collagen-based biomaterials showed higher passive and active twitch force, aligned sarcomeres, regularly dispersed connexin-43/N-cadherin, and increased expression of maturation markers^48^. However, the metabolic changes in the cells are rarely considered, possibly because metabolism in larger 3D constructs cannot easily be quantified using the Seahorse Bioanalyser, which is the strategy used most frequently to quantify metabolic changes.

We found that both maturation methods induced a degree of maturation with reduced glycolysis and increased FAO in the hiPSC-CMs but the effect of 3D culture was dominant. We hypothesized that the hiPSC-CMs were unlikely to upregulate FA oxidation-associated pathways while being cultured in media containing little to no FAs. We saw a decrease in glycolysis, after 1 week of treatment of cells grown in 2D with OA, with no change in glucose oxidation and an increase in palmitate oxidation. However, the relative rates of glycolysis and palmitate oxidation meant that the cells retained a predominantly glycolytic phenotype. In contrast, treatment with both OA and WY-14643 did not change rates of glycolysis or of glucose oxidation but did further increase rates of FAO, so that overall rates of energy generation increased. Interestingly, we found that baseline rates of palmitate oxidation were lower than those of oleate but that, whether hiPSC-CMs were cultured in 2D or 3D, addition of OA ± WY-14643 to the culture medium increased rates of palmitate oxidation but not those of oleate. Saturated and unsaturated fatty acids are known to have different roles in cellular processes^56^ and work in the isolated perfused heart has shown that OA is more readily stored as triglycerides than palmitate, resulting in increased triglyceride turnover with OA than palmitate^57^. Our measurement of fatty acid oxidation, using ^3^H-labeled substrates, works by quantifying release of ^3^H_2_O during beta-oxidation. ^3^H-OA that is incorporated in endogenous triglyceride would not be detected by this process, nor would beta-oxidation of ^1^H_2_O liberated from triglyceride stores during turnover. Therefore, it could be that oleate is being preferentially diverted via storage as triglycerides, but further research is needed to be prove this hypothesis.

3D culture on collagen-derived scaffolds accelerated the structural and metabolic maturation and increased mitochondrial oxidative metabolism while decreasing anaerobic glycolysis. This was associated with changes in the expression of mRNA of structural proteins and of *PGC1α*, associated with mitochondrial biogenesis, and *PDK4*, which controls the switch between glycolysis and glucose oxidation. Interestingly, the combination of 3D culture with PPARα activation did not have an additive effect, except when the hiPSC-CMs were grown on the collagen/elastin scaffolds where we saw a similar decrease in glycolysis with OA but not with OA + WY-14643 to that we saw in monolayer cells (albeit not significant) and an increase in the rates of both glucose and palmitate oxidation. It is likely that, as was seen in the study by Rana et al.^53^, the increased energy generation induced by either method was sufficient for the hiPSC-CMs to contract, such that a further increase in ATP was not necessary. Where the cells were grown on the collagen/elastin scaffold, the increased elasticity^31^ may have enabled the hiPSC-CMs to increase the rate of contraction when provided with additional ATP via oxidative metabolism. In proliferative cells, glycolysis and glucose oxidation are uncoupled, with high rates of glycolysis under aerobic conditions, whereas in the adult heart glycolysis and glucose oxidation act in synergy to maximize ATP generation^58^. The increase in glucose oxidation seen where hiPSC-CMs were cultured with OA and in 3D on collagen/elastin scaffolds suggests that increased maturation, induced by the combination of structural and metabolic stimulation, resulted in increased coupling between glucose oxidation and glycolysis.

Recent work by Ulmer et al.^32^ showed, using radiolabeled substrate measurements, that culture of hiPSC-CMs in EHTs led to the maturation of energy metabolism when compared to 2D culture. The EHTs had an increase in mitochondrial mass and in glucose, lactate, and FA oxidative metabolism with an associated reduction in anaerobic glycolysis. These results strongly support the notion that contractile work contributed to the metabolic maturation of hiPSC-CMs. However, this study only looked into the use of 3D culture and mechanical load/stimulation to induce maturation and did not target any modification of the medium. It is noteworthy that the EHT medium contains 10% horse serum which contains around 0.5 μmol/mL of mixed fatty acids^59^. This results in a final medium concentration of 50 μM FA, before addition of 400 μM oleate, compared to the 2 μM in B27 used routinely in normal 2D and scaffold culture. As shown by Ulmer et al.^32^*,* we saw a significant switch from glycolysis to FAO induced by growing the cells in EHT. The rate of palmitate oxidation was further increased by addition of OA and WY-14643 for 1 week such that the cells oxidized 20 times the amount of palmitate used by the untreated hiPSC-CMs grown in 2D. We did not detect changes in sarcomere structure or mitochondrial membrane potential as a result of the addition of OA and WY-14643, although there was a significant increase in mRNA expression for *PGC1α* and *MYH6*. In the adult heart the utilization of one nutrient directly inhibits the use of the other via the Randle cycle, and FAO oxidation generates substantially more ATP than is produced by glycolysis^1^. The substrate switch in treated EHTs may therefore be associated with a maturation of pathways that feed into the mitochondria. and suggest a metabolic flexibility that more closely matches that of the adult human heart.

The metabolic changes induced by modulating the culture medium and by 3D culture have been investigated by others. Horikoshi et al. used a glucose-free maturation medium containing linoleic acid and OA in 2D hiPC-CMs for 3 to 7 days and saw structural maturation and increased glycolysis and oxidation of PA^23^ whereas with addition of OA to glucose-containing media we saw decreased glycolysis after 7 days. They suggested that the increased glycolytic capacity may indicate that the cells have a higher capacity to switch to glucose oxidation when FAO is compromised. Yang et al. showed that addition of physiological amounts of PA, OA and linoleic acids increased structural maturation, glucose oxidation and calcium handling of 2D hiPSC-CM but they did not measure FAO directly^21^. Mills et al. investigated maturation in ‘Heart-Dyno’, contractile organoids in a 96-well plate format which, like EHT, contract against elastic posts^24^. They used low glucose medium supplemented with 10 or 100 μM palmitate and saw increased force of contraction and expression of cardiac proteins. Using the Seahorse XF Bioanalyzer and metabolomic measurements, they confirmed a switch from glycolysis to fatty acid oxidation. Interestingly, they found that increased palmitate oxidation was linked to inhibition of key proliferation pathways and that insulin counteracted this effect. Clearly careful tailoring of maturation media is required, as palmitate has been shown to increase apoptosis in AC16 cardiomyocytes^60^ and high concentrations of palmitate and insulin have been used induce an ‘insulin resistant’ phenotype in contracting mouse HL-1 cells^61^. Most recently, Feyen et al. used a maturation medium containing low glucose with lactate and a mixture of albumin-bound fatty acid (AlbuMAX), creatine, L-carnitine and taurine to support CM energetics^54^. They showed metabolic, structural and electrophysiological maturation in 2D and enhanced sarcomere structure, contraction and survival of EHTs with this maturation medium. Thus, different approaches to iPSC-CM maturation are being investigated and are beginning to provide a picture of the interplay between metabolic, functional and structural maturation. Further work is required to determine whether the cells have also achieved the metabolic flexibility of the adult heart.

## Conclusions

We have directly compared glucose utilization and saturated and unsaturated FAO in hiPSC-CM cultured in 2D and 3D with manipulation of the culture media. The switch to a more metabolic phenotype in hiPSC-CMs, with increased FAO, can be induced by culture in 3D and enhanced by pharmacological stimulation of the PPARα pathway. However, the two approaches are not necessarily additive if the energy requirement of the cells does not change substantially. In EHT, the cells are under an increased workload and can therefore benefit from further upregulation of FAO. Changing metabolism is only one aspect of cardiac maturation in vivo*,* which results from many factors. Future steps could involve combinations of strategies such as 3D culture, electrical stimulation, force, and metabolic maturation. Maturation media have been building traction^20,21,23,24,53,54,62^ and may prove to be a solid cornerstone to enhancing maturation by better mimicking in vivo conditions. Targeting maturation from multiple sides will hopefully be the edge needed to generate an ideal phenotype to model human adult CMs.

## Materials and methods

More detailed methods can be found in the Supplementary.

### hiPSC-CMs differentiation

hiPSCs (OX1-19) grown in mTeSR1 media on Matrigel-coated flasks, were dissociated using TrypLE Express or ReLeSR and transferred to flasks coated with Growth Factor Reduced Matrigel (RF-Matrigel) for differentiation into beating CMs. Cells were sandwiched with RF-Matrigel. To commence differentiation, media was changed to Roswell Park Memorial Institute (RPMI) 1640 supplemented with 1% B27 minus insulin (RPMI-B27), 12 μM CHIR99201, 10 ng/mL Activin A, and 1:200 RF-Matrigel. After 24 h (Differentiation Day 1, DD1), the media was changed to RPMI-B27 with no additional factors. On DD3, half of the media was changed to fresh RPMI-B27 with the addition of 5 μM IWP4 or 2.5 µM Wnt-C59. On DD5, the medium was changed again to RPMI-B27 with no additional factors. On DD7, the media was changed to RPMI 1640, containing 1% B27 Complete (RPMI + B27) and the flasks were cultured under normoxia. From this point on, the culture media was changed every 2–3 days.

### Collagen-derived scaffolds

Scaffolds were prepared using 100% type I collagen (from bovine achilles tendon) or a 50:50 mixture of type I collagen with chondroitin-4-sulfate (C4S) (from bovine trachea) or elastin (from bovine neck ligament). Suspensions at 1% wt/v were freeze-dried, as previously described^30, 31^. The suspensions were degassed under a vacuum of 10 Pa and cast into polytetrafluoroethylene moulds to form disc-shaped scaffolds (diameter: 13 mm, height: 4 mm). The moulds were frozen at − 20 °C, and then freeze-dried (Christ I-5, Martin Christ) for 24 h in a vacuum of 5 Pa^30,31^.

### Seeding scaffolds

The collagen-based scaffolds were sterilised by submersion in 100% ethanol (3 × 30 min) and washed with Dulbecco’s Phosphate Buffered Saline (DPBS) containing antibiotics and then RPMI + B27. hiPSC-CMs were dissociated and counted. Each scaffold was placed in a well of a 24-well plate and loaded with 10^6^ cells in 600 μL of media containing rock inhibitor (Y-27632, 10 μM). The media was changed the next day and every subsequent 2–3 days by manually picking up the scaffold with sterilized tweezers and transferring it to a new well containing 1.5 mL of fresh media.

### Engineered heart tissue generation

EHTs were generated using the protocol as previously described^63,64^. Briefly, hiPSC-CMs were differentiated and dissociated on DD15 into single cells. For a single EHT, 10^6^ hiPSC-CMs were re-suspended in 92 μL of NKM media (Dulbecco’s Modified Eagle Media (DMEM) incl. 1% penicillin–streptomycin (P/S), 10% fetal calf serum inactive, 1% glutamine), 5.5 μL 2 x DMEM (20% 10 x DMEM (670 mg DMEM in 5 mL ddiH_2_O), 20% horse serum inactive, 1% P/S in sterile water), 0.425 μL aprotinin, 3 μL bovine thrombin and 2.5 μL of fibrinogen, pipetted into a pre-cast 2% agarose mould prepared using Teflon spacers and solidified around two silicone posts over a 3-h span.

### Treatment with oleate and WY-14643

hiPSC-CMs were re-plated on DD15 into a 2D RF-Matrigel-coated 24-well plate, seeded onto a 3D collagen-derived scaffold, or encapsulated in an EHT and treated with control medium or medium containing 400 μM OA (pre-conjugated to Bovine Serum Albumin [BSA]) optionally with 120 μM WY-14643 for up to 1 week, with media refreshed every other day. Untreated hiPSC-CMs were used as controls.

### Quantitative PCR

Total ribonucleic acid (RNA) was extracted using a Qiagen RNeasy Kit according to the manufacturer's protocol. Scaffolds were digested by incubation in TrypLE Express for 3 min at 37 °C. RLT lysis buffer supplemented with β-mercaptoethanol was added and scaffolds were shredded and the processed using the Qiagen RNeasy Kit. The RNA concentration was determined using NanoDrop UV spectrometry and 1 μg of RNA was converted into complementary deoxyribonucleic acid (cDNA) using a high capacity cDNA reverse transcriptase kit according to the manufacturer's protocol. The samples were incubated in a SensoQuest Basic Thermal Labcycler and qPCR was performed using a StepOnePlus Real-Time PCR System, either with the SYBR detection method or TaqMan Gene Expression Assay. Primers are listed in Tables S1 and S2. Data were analysed using the ΔΔCT method^65^, plotting the fold change of 2^−ΔΔCT^.

### ^3^H Palmitate and oleate oxidation

FAO rates were determined by the production of ^3^H_2_O in the electron transport chain as previously described^66,67^. For palmitate oxidation the cells were cultured with 1.5 mL of the RPMI + B27 containing 2% BSA, 0.3 mM palmitate (added to heated media for conjugation to BSA), and 0.2 μCi/mL of palmitate, [9,10-^3^H(N): 1 mCi-37 MBq] per well of a 24-well plate. For oleate oxidation, the cells were cultured with 1.5 mL of RPMI + B27 containing 2% BSA, 400 μM OA, and 0.2 μCi/mL of oleate, [9,10-^3^H(N): 1 mCi-37 MBq]. Cells were cultured in an incubator (5% CO_2_ and 21% O_2_) at 37 °C for 8 h. The ^3^H_2_O released into the supernatant was separated from the remaining ^3^H-palmitate or ^3^H-OA via Folch extraction. The top aqueous layer (0.5 mL in duplicate) was added to 10 mL of Ecolite liquid scintillation cocktail and radioactivity was counted using a Tri-Carb 2800TR Liquid Scintillation Analyzer. Rates of oxidation were calculated as described in ^67^.

### ^3^H glycolysis

Glycolytic rates were determined through the conversion of ^3^H-glucose to ^3^H_2_O via enolase. The cells were cultured with 1.5 mL of RPMI-low glucose media (5.5 mmol/L) and 0.2 μCi/mL of Glucose, D-[5-^3^H(N)]: 1 mCi-37 MBq] per well of a 24-well plate, in an incubator (5% CO_2_ and 21% O_2_) at 37 °C for 8 h. The supernatant was collected and the ^3^H_2_O was separated from the ^3^H-glucose using a Dowex anion exchange column. The radioactivity was counted as above and rates of glycolysis calculated as described in ^67^.

### ^14^C glucose oxidation

Glucose oxidation were determined through the use of a standard ^14^CO_2_ capture technique ^66,68^. Cells in a 24-well plate were incubated in 1 mL of RPMI-low glucose media doped with 0.2 μCi/mL of Glucose, [D-[^14^C(U): 1 mCi-37 MBq]. Released ^14^CO_2_ was trapped on potassium hydroxide-soaked filter papers. The ^14^C capture apparatus was sealed and placed inside an incubator (5% CO_2_ and 31% O_2_) for 8 h. 1 mL of 70–72% perchloric acid was then added and the samples kept for 1–2 h for complete release of dissolved ^14^CO_2_. Filter papers containing trapped ^14^CO_2_ were placed into 10 mL aliquots of Ecolite liquid scintillation cocktail and counted as above.

### Oxygen consumption rate measurements using the Clark-type oxygen electrode

The rate of mitochondrial oxygen consumption (OCR) was measured using the Clark-type oxygen electrode^69,70^. Cells were rinsed with DPBS and dissociated into single cells. Two million cells were centrifuged (1300 rpm, 4 min), supernatant was discarded, and the cell pellet was resuspended in 600 μL of freshly prepared respiration solution (100 mM potassium chloride, 50 mM 3-(N-morpholino)propanesulfonic acid, 1 mM ethylene glycol-bis(β-aminoethyl ether)-N,N,N′,N′-tetraacetic acid, 5 mM KH_2_PO_4_ and 1 mg/mL BSA) or the cell culture media. The oxygen electrodes were embedded in the reaction chambers set to 37 °C. 600 μL of respiration solution containing cells were transferred into the respiratory chambers. Respiration was measured under baseline conditions with the addition of the energy substrate of interest (10 mM pyruvate + 5 mM malate, or 400 μM oleate) and after addition of the metabolic uncoupler, carbonylcyanide-p-triuoromethoxy-phenylhydrazone (10 mM FCCP).

### Flow cytometry

hiPSC-CMs were dissociated to a single-cell suspension with TrypLE Express and incubated in Zombie Violet. The cells were spun down and fixed with 4% paraformaldehyde (PFA). After incubation with blocking media, samples were incubated with monoclonal anti-actinin sarcomeric (diluted 1:200 in blocking media) for 1 h at room temperature (RT), or overnight at 4 °C, washed and incubated with Alexa Fluor 594; donkey anti-mouse (1/200) for 1 h at RT. After further washing, each sample was resuspended in 0.6–1 mL of DPBS and analysed using a BD Fortessa X20 cell analyzer. 30,000 events were acquired for each sample and the data was analyzed with FlowJo Software 10.0.

### Immunocytochemistry

The cells were washed with DPBS, fixed in 4% PFA and washed with DPBS. Fixed cells were permeabilized by incubation with 0.05% Triton-X for 10 min at RT, washed with DPBS and incubated in blocking media (DPBS, 2% BSA) for 60 min at RT. The samples were incubated with monoclonal anti-actinin sarcomeric (1:500 in blocking media) for 1 h at RT, or overnight at 4ºC, washed with DPBS, and incubated with Alexa Fluor 594; donkey anti-mouse (1/200) for 1 h, in the dark at RT. The sample was washed twice with DPBS. DAPI stain (diluted in DPBS) was added for 5 min at RT, and the slides were rinsed in DPBS and mounted with 50:50 DPBS/glycerol. Images were taken using an Olympus Confocal Microscope.

### MitoTracker staining

hiPSC-CMs were incubated in RPMI or non-FBS medium with 100 nM of MitoTracker Red CMXRos dye for 40 min at 37 °C. The cells were washed with DPBS and fixed in 4% PFA for 20 min at 4 °C. Fixed cells were washed with DPBS with further staining as described above and imaged using an Olympus Confocal Microscope. ImageJ was used to analyze the fluorescence intensity of MitoTracker Red CMXRos dye. The level of fluorescence of randomly selected cells was analyzed by measuring the grey value of stained areas and unstained areas (background). Fluorescence intensity was calculated by taking the average of 10 mean grey values of selected cells minus the mean fluorescence of background.

### Scanning electron microscopy (SEM)

SEM images of the samples were obtained using the Zeiss EVO MA10 (10 kV) to determine the scaffold and cell morphology. Scaffolds were seeded on DD15 and cultured for 1 week before fixation with 4% PFA for 30 min at RT. Fixed cells were washed with deionised water, frozen and freeze-dried. Freeze-dried scaffolds (non-seeded and seeded) were sputter-coated in gold (using a Bio Rad E5400) prior to SEM analysis.

### Statistical analysis

All experimental samples were run in triplicate with at least three biological repeats. Gene expression and metabolism data are presented as means ± standard errors. Differences between samples were considered significant if *p* < 0.05 as determined using either a Student's T-test or a one-way Analysis of Variance (ANOVA). Experiments with multiple variables and test groups were assessed using a two-way ANOVA with a Tukey post-hoc test in GraphPad Prism 7.

## Supplementary Information

Supplementary Information 1.
