# Supplementary material for: Physiological and pharmacological stimulation for in vitro maturation of substrate metabolism in human induced pluripotent stem cell-derived cardiomyocytes
Source: Sci Rep. 2021 Apr 8;11:7802. doi: 10.1038/s41598-021-87186-y (PMC8032667; doi:10.1038/s41598-021-87186-y)
Supplement: Supplementary file 1 — Supplementary Information 1. [file 41598_2021_87186_MOESM1_ESM.pdf]

Physiological and pharmacological stimulation for *in vitro* maturation of substrate metabolism in human induced pluripotent stem cell-derived cardiomyocytes.

Colleen A. Lopez*[1, 3], Heba Hussain A. A. Al-Siddiqi*[1, 4], Ujang Purnama[1], Sonia Iftekhar[2], Arne A. N. Bruyneel[1], Matthew Kerr[1], Rabia Nazir[2, 5,] Maria da Luz Sousa Fialho[1], Sophia Malandraki-Miller[1], Rita Alonaizan[1], Fatemeh Kermani[1], Lisa C Heather[1], Jan Czernuszka[2], Carolyn A Carr[1#]

[1]Department of Physiology, Anatomy and Genetics, University of Oxford, Sherrington Building, Parks Road, Oxford, UK, OX1 3PT;

[2]Department of Material Science, University of Oxford, Parks Road, Oxford, UK, OX1 3PH

[3]School of Medicine's Cardiovascular Medicine Research Center, Yale University, 300 George Street, New Haven, CT 06511, USA

[4] Qatar Biomedical Research Institute, Hamad Bin Khalifa University, P.O. Box: 34110

Education City, Doha, Qatar

[5]Interdisciplinary Research Centre in Biomedical Materials, COMSATS University Islamabad, Lahore Campus, 1.5 KM Defence Road Off Raiwind Road, Lahore, Pakistan

* These authors contributed equally.

# Corresponding Author: Professor Carolyn Carr; Department of Physiology, Anatomy and Genetics, Sherrington Building, Parks Road, Oxford, UK, OX1 3PT

Email Carolyn.carr@dpag.ox.ac.uk

Telephone 0044 1865 282247

Orchid ID 0000-0003-0102-5261

Supplementary Figures:

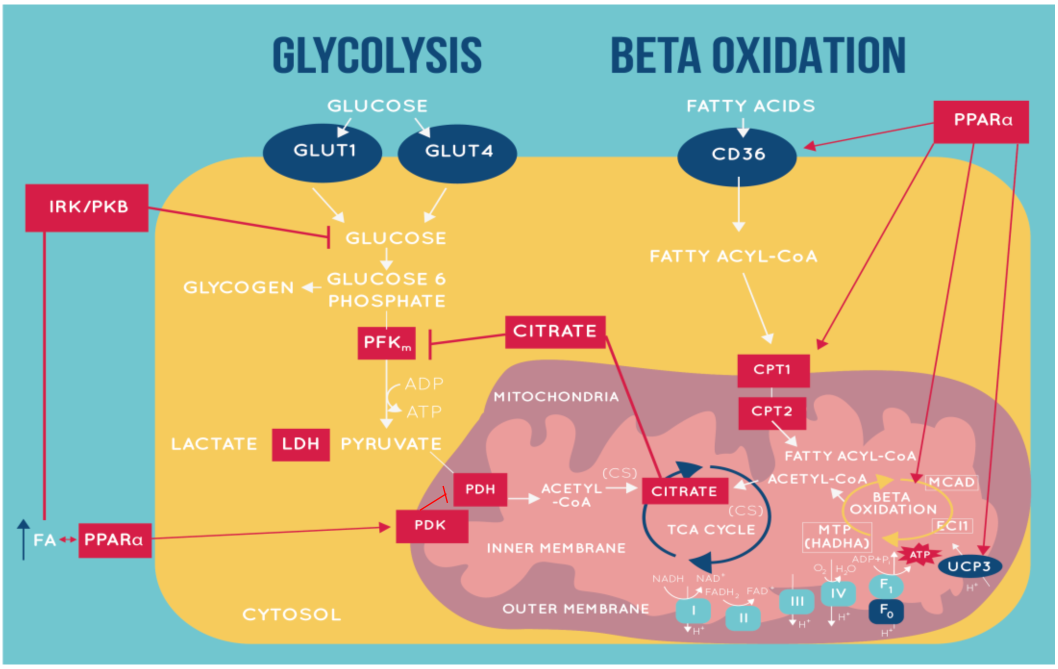

Figure S1: Schematic showing key proteins involved in glycolysis and β-oxidation

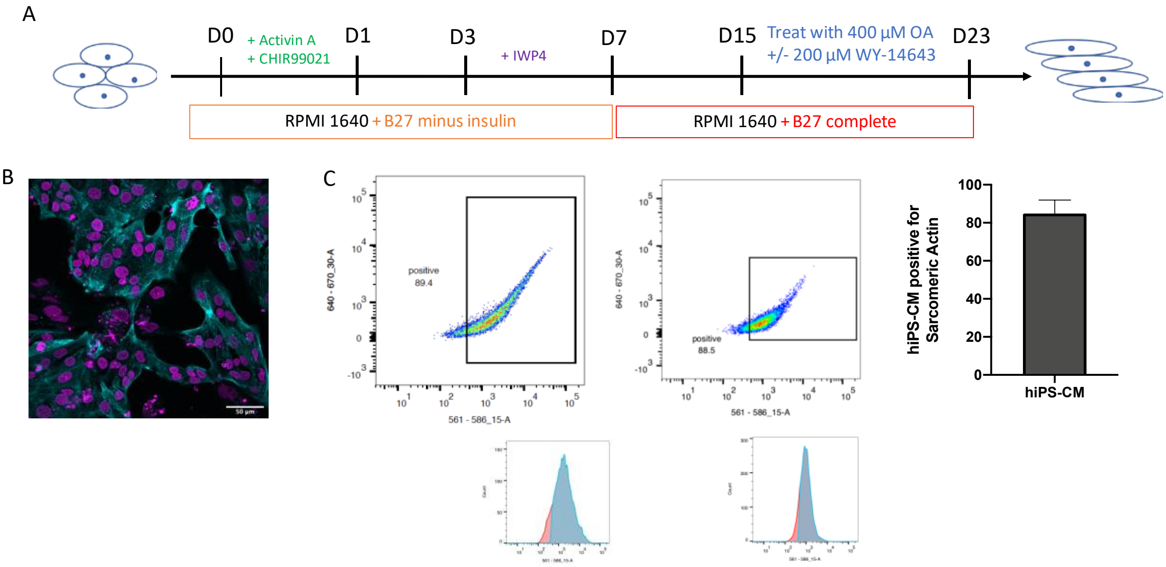

Figure S2: A) Schematic of hiPSCs differentiation into beating CMs. Immunostaining B) and flow cytometry analysis C) for α-Sarcomeric Actinin (Actinin-cyan, DAPI-magenta) expression in beating hiPSC-CMs on DD15.

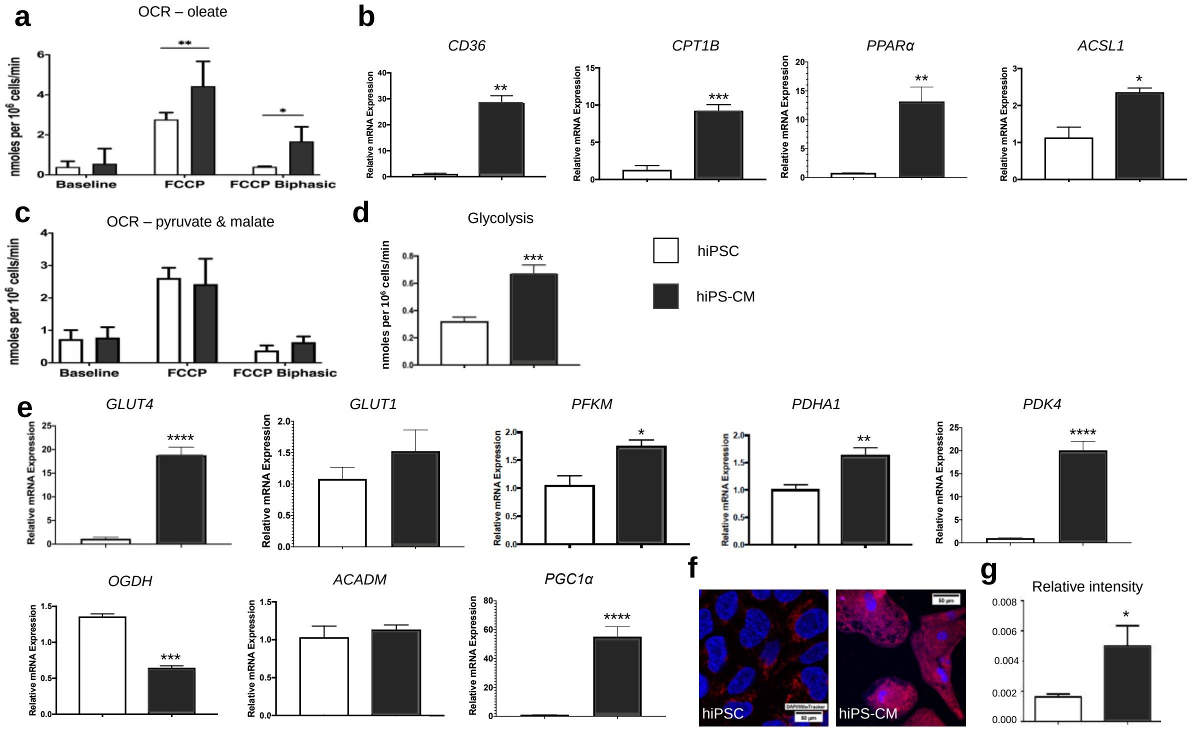

Figure S3: hiPSC were differentiated into iPSC-CMs over 1 month and changes measured in a) oxygen consumption rate (OCR) when respiring on oleate (n=4); b) mRNA expression of genes involved in fatty acid oxidation (n=3); c) OCR when respiring on pyruvate and malate (n=4); d) rate of glycolysis (n=4); e) mRNA expression of metabolic genes (n=3); and f) mitochondrial staining using Mitotracker CMXRos, quantified in (g); *, **, ***, **** $p < 0.05, 0.01, 0.001, 0.0001$ compared with hiPSC.

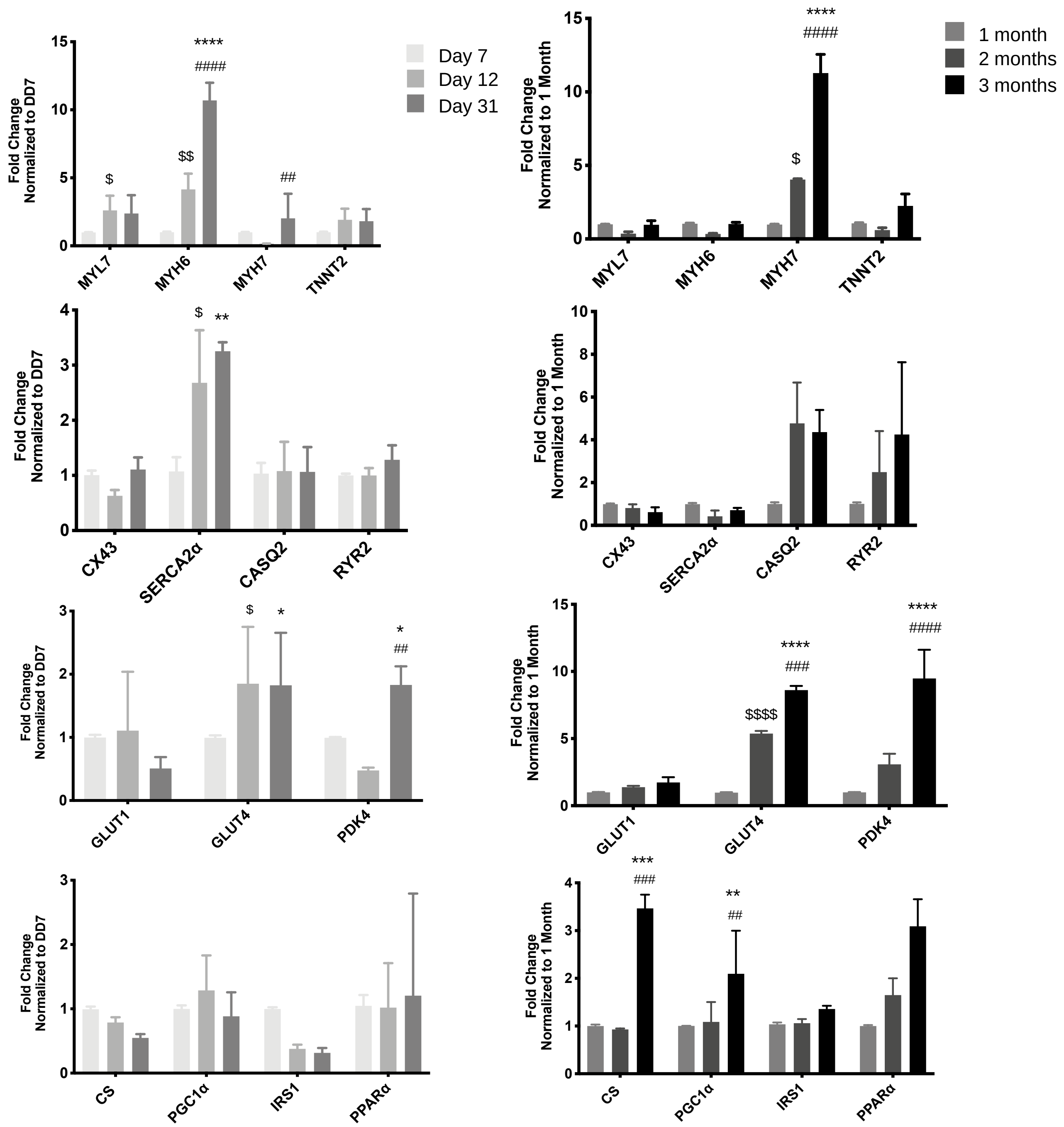

Figure S4. Changes in mRNA expression of cardiac, metabolism and calcium handling genes in iPSC-CMs grown in monolayer culture and harvested after day 7, 12 and 31, and 2 and 3 months of differentiation. Left panel are normalized to day 7, while right panel to 1 month. *, **, ***, **** $p < 0.05, 0.01, 0.001, 0.0001$ compared with day 7/1 month respectively; #, ##, ###, #### $p < 0.05, 0.01, 0.001, 0.0001$ compared with day 12/2 months respectively (n=3-6).

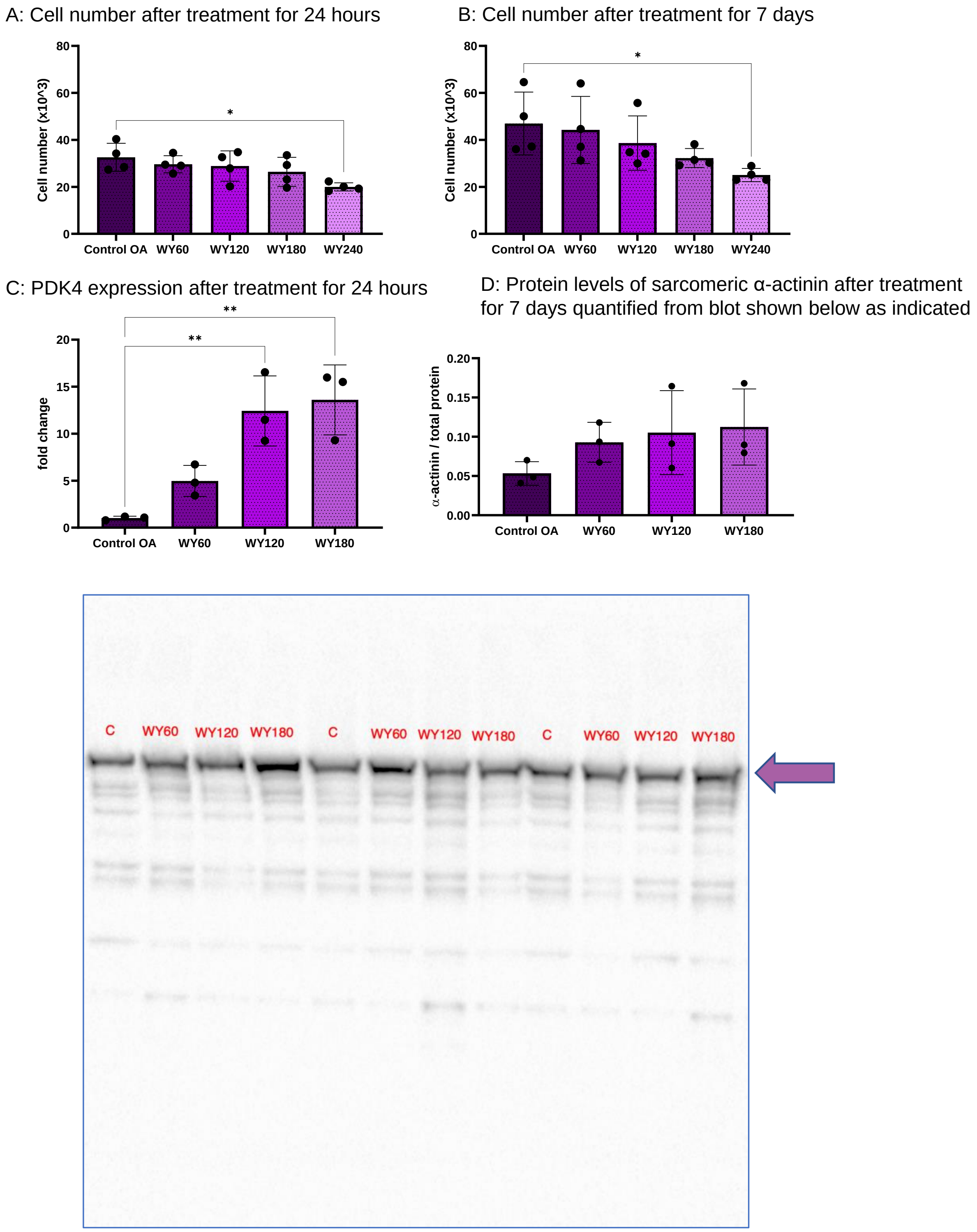

Figure S5: Dose response to WY14643: hiPSC-CM were treated with 400 μM OA alone or with 60, 120, 180 or 240 μM WY14643 for 24 hours (A) and 7 days (B). PDK4 mRNA expression was tested after treatment with OA ± 60, 120 or 180 μM WY14643 for 24 hours (C) and protein levels of sarcomeric α-actinin were assessed by Western blotting after treatment for 7 days (D) *, ** $p < 0.05$ and 0.01, compared with control OA-treated cells (n = 3 or 4 as indicated).

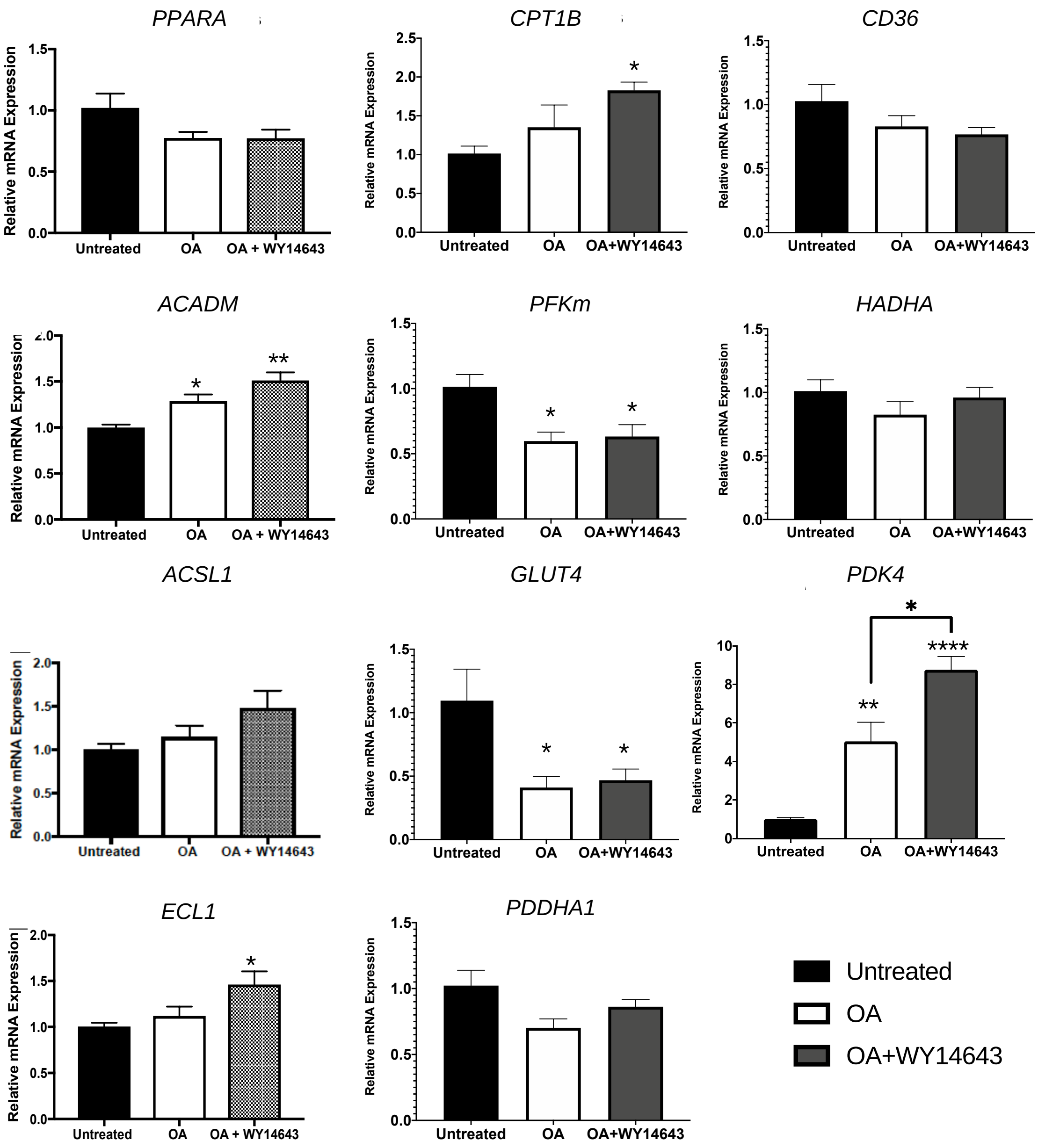

Figure S6: Changes in mRNA gene expression of hiPSC-CMs cultured as a monolayer on RF-Matrigel respectively for 8 hours with OA ± WY-14643; *,**,***,**** = P<0.05, 0.01, 0.001, 0.0001 respectively compared with untreated hiPSC-CMs; #,##,###,#### = P<0.05, 0.01, 0.001, 0.0001 compared with OA treated cells.

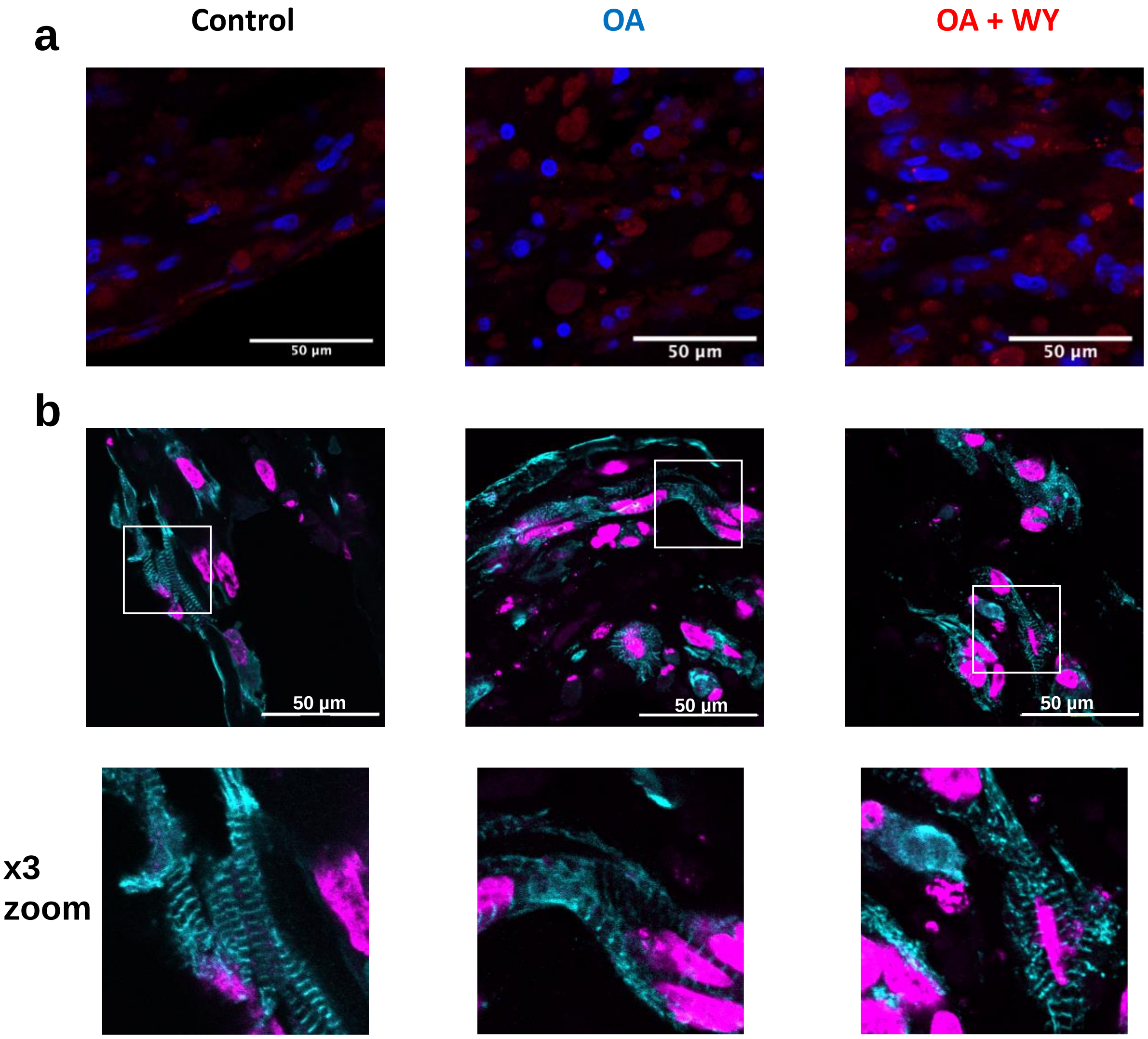

Figure S7: Images taken from a confocal microscopy z-stack of control and treated EHT, after 1 week of treatment with OA +/- WY-14643, stained with (a) MitoTracker Red CMXRos dye to show mitochondrial membrane potential and (b) an antibody for α-sarcomeric actinin showing sarcomeres in the cardiomyocytes. Scale bars in (a) and (b) are 50 µm, with a x3 zoom on (b) shown below.

Supplementary Methods

hiPSC-CMs differentiation: hiPSC-CMs differentiation: hiPSCs (OX1-19) grown in mTeSR1 media (STEMCELL Technologies) on Matrigel-coated flasks (Corning), were dissociated using TrypLE Express (Gibco) or ReLeSR (STEMCELL Technologies) and transferred to Growth Factor Reduced (RF) Matrigel-coated flasks (Corning) diluted 1/100 in DMEM/F12 (Gibco) at 384k cells cm^2 for differentiation into beating CMs. The following day (DD0), the media was changed to Roswell Park Memorial Institute (RPMI) 1640 (ThermoFisher), supplemented with 1% B27 minus insulin (ThermoFisher), 12 µM CHIR99201 (BioTechne), 10 ng/mL Activin A (RD Systems Europe Ltd) and 1:200 RF-Matrigel. After 24 hours (DD1), the media was changed to RPMI 1640 with 1% B27 minus insulin with no additional factors. On DD3, half of the media was changed to fresh RPMI 1640 containing 1% B27 minus insulin with the addition of 5 µM IWP4 (tebu-bio) or 2.5 µM Wnt-C59 (Bio-Techne). On DD5, the medium was changed again to RPMI 1640 with 1% B27 minus insulin with no additional factors. On DD7, the media was changed to RPMI 1640, containing 1% B27 Complete (ThermoFisher). From this point on, the culture media was changed every two-three days.

Collagen-derived scaffolds: Type I collagen (from bovine achilles tendon), chondroitin-4-sulfate (C4S) (from bovine trachea) and elastin (from bovine neck ligament), were selected as scaffold materials and were all obtained from Sigma-Aldrich Ltd, UK. Scaffolds were fabricated by freeze-drying suspensions with various compositions, as required and previously described (Chen et al., 2012, 2013). For collagen and collagen-elastin scaffolds, a suspension was made with the desired weight ratio (50% collagen-50% elastin) in 0.05 M acetic acid (pH 3.2). All the suspensions were of the same total concentration of 1% weight by volume (wt/v). For the 50% collagen-50% C4S scaffolds, collagen was first suspended in acetic acid, then the C4S was added into the suspension, and instantly the C4S co-precipitated with the collagen forming an agglomeration. The suspension was reduced by drop-wise addition of 2M sodium hydroxide until the pH value reached 7. This resulted in a homogeneous suspension of very fine collagen I-C4S co-precipitates. The total concentration of the suspension was 1% wt/v. The suspensions were degassed under a vacuum of 10 Pa before they were cast into polytetrafluoroethylene (PTFE) moulds to form disc-shaped scaffolds (diameter: 13 mm, height: 4 mm). Next, the moulds were frozen at -20 ◦C, and then freeze-dried (Christ I-5, Martin Christ) for 24 hours in a vacuum of 0.05 mbar. The ice crystals were removed by sublimation, leaving behind the porous solid scaffolds.

Seeding Scaffolds:
The collagen-derived scaffolds were sterilised by submerging them in 100% ethanol (3 x 30 minutes), followed by washing (3 x 30 minutes) with DPBS containing antibiotics (100 U/mL penicillin, 0.1 mg/mL streptomycin (P/S) and 0.25 µg/mL amphotericin B, Sigma-Aldrich Ltd). A final wash was conducted in RPMI 1640 with B27 Complete supplement before scaffolds were seeded with the cells. hiPSC-CMs were dissociated as described earlier, counted and then seeded onto different collagen-derived scaffolds. After sterilisation, each scaffold was placed in its own well of a 24-well plate. The top section of a 1 mL pipette tip was cut off and placed vertically on the scaffold to act as a mould (Figure 3A). When the scaffold was moist from the washes, the tip attached by suction to the scaffold and stood unassisted. Each scaffold was then loaded with 10^6 hiPSC-CMs in 600 µL of media containing rock inhibitor (10 µM Y-27632, Merck Chemicals Ltd). The plate was then placed in the incubator for 5 hours, at which point the tips were removed and the well was loaded

with an additional 500 µL of RPMI 1640 with B27 Complete supplement with P/S. The media was changed the next day and every 2-3 days from then by manually picking up the scaffold with sterilized tweezers and transferring it to a new well containing 1.5 mL of fresh media.

Engineered Heart Tissue (EHT) generation: EHTs were generated using the protocol as previously described (Breckwoldt et al., 2017; Hansen et al., 2010). Briefly, hiPSC-CMs were differentiated and dissociated on DD15 into single cells. For a single EHT, 10^6 hiPSC-CMs were re-suspended in 92 µL of NKM media (DMEM incl. 1% P/S [Gibco], 10% fetal calf serum inactive (ThermoFisher), 1% Glutamine (Life Technologies)), 5.5 µL 2xDMEM (20% 10xDMEM (670 mg DMEM in 5 mL ddiH_2O), 20% horse serum inactive [Gibco], 1% P/S in sterile water), 0.425 µL aprotinin (Sigma-Aldrich Ltd), 3 µL bovine Thrombin (Biopur), and 2.5 µL of Fibrinogen (Sigma-Aldrich Ltd). Each mixture was then pipetted into a pre-cast 2% agarose mould (prepared using Teflon spacers: EHT Technologies) and solidified around two silicone posts (EHT Technologies) over a 3-hour span.

Treatment with oleate and WY-14643: hiPSC-CMs were treated with oleate ± WY-14643 to enhance maturation. The hiPSCs were differentiated into CMs and re-plated (10^6 hiPSC-CMs) on DD15 into a 2D RF-Matrigel-coated 24-well plate, seeded onto a 3D collagen-derived scaffold, or encapsulated in an EHT. In each case, cells were treated with control medium or medium containing 400 µM OA (pre-conjugated to BSA; Sigma-Aldrich Ltd) ± 120 µM WY-14643 (Selleck Chemicals) for up to 1 week, with media refreshed every other day for 1 week. The untreated hiPSC-CMs were used as controls for all the experiments.

CCK-8 Assay: hiPSC-CM were seeded in a 96-well plate and treated with WY-14643 for 24 hours or 7 days. At the end of the experiment, CCK-8 solution (10 uL) was added to each well and incubated for 2 hours. The absorbance was read at 450 nm and cell number calculated using a standard curve.

Quantitative PCR: Total ribonucleic acid (RNA) was extracted using a Qiagen RNeasy Kit (Qiagen) according to the manufacturer's protocol. Scaffolds were digested by incubation in TrypLE Express for 3 minutes at 37°C. RLT lysis buffer (Qiagen) supplemented with 14.3 M β-mercaptoethanol (Sigma-Aldrich Ltd; 1 µL BME per 1 mL RTL Buffer) was added and scaffolds were shredded using a QIA shredder gun, prior to being processed further using the Qiagen RNeasy Kit. The RNA concentration was determined using NanoDrop UV spectrometry (NanoDrop TM Lite, Thermo Fisher Scientific), and 1 µg of RNA was converted into complementary deoxyribonucleic acid (cDNA) using a high capacity cDNA reverse transcriptase kit (Applied Biosystems) according to the manufacturer's protocol. The samples were then incubated in a SensoQuest Basic Thermal Labcycler (SensoQuestGeneFlow): 10 min at 25°C, 120 min at 37°C, and 5 min at 85°C. qPCR was performed using either the SYBR detection method (Applied Biosystems; primers listed in Table S1) or TaqMan Gene Expression Assay (Invitrogen) and TaqMan Fast Advanced PCR Master Mix (2x) (Invitrogen). The samples were loaded in a 96-well format qPCR plate (MicroAmp Fast, Applied Biosystems TM, China), and a standard SYBR cycle was started using a StepOnePlus Real-Time PCR System (Applied Biosystems). For the TaqMan assay, amplification was carried out using TaqMan Fast Advanced PCR Master Mix (2x) (Invitrogen) according to the supplier's protocol. It involved a 10 µL reaction using 12.5 ng of cDNA, ddH_2O, and the TaqMan assays (Table S2). Data were analysed using the ΔΔCT method (Livak and Schmittgen, 2001), plotting the fold change of $2^{-\Delta\Delta CT}$.

^{3}H Palmitate and Oleate Oxidation: FAO rates were determined by the production of $^{3}H_2O$ in the electron transport chain as previously described (Barr RL, 2000; Lopaschuk and Barr, 1997). For palmitate oxidation the cells were cultured with 1.5 mL of the RPMI 1640 with B27 Complete supplement containing 2% BSA, 0.3 mM palmitate (added to heated media for proper conjugation to BSA), and doped with 0.2 µCi/mL of Palmitic Acid, [9,10-^{3}H(N): 1mCi-37MBq] (PerkinElmer) per well of a 24-well plate (1.5 mL per 1.9 cm^2 growth area). For oleate oxidation, the cells were cultured with 1.5 mL of the RPMI 1640 with B27 Complete supplement containing 2% BSA, 400 µM OA, and doped with 0.2 µCi/mL of oleate, [9,10-^{3}H(N): 1mCi-37MBq] (PerkinElmer). Cells were cultured in a normoxic incubator (5% CO_2 and 21% O_2) at 37°C for 8 hours. During the incubation, $^{3}H_2O$ was released into the incubation media. After the incubation, the $^{3}H_2O$ in the supernatant was separated from the remaining ^{3}H-palmitate or ^{3}H-OA via Folch extraction. A volume of 1.88 mL of chloroform: methanol (1:2 v/v) solution, 625 µL chloroform and 625 µL KCl-HCl solution (2M KCl, 0.4 M HCl) was added to 0.5 mL of perfusate sample. The mixture was rotated on a laboratory Stuart rotator SB3 at 40 rpm for 10 minutes and left in the hood for 1 hour to allow the different layers to separate. The top aqueous layer was then removed with a glass pipette into a new 15 mL falcon tube while the organic (bottom) layer was discarded. To the extracted aqueous solution, 1 mL chloroform was added, with 1 mL methanol and 0.9 mL KCl-HCl solution before mixing by rotation for another 10 minutes at 40 rpm and being left to separate for 1 hour. The top aqueous layer was retained, and 0.5 mL (in duplicate) of this phase was added into a scintillation vial containing 10 mL of Ecolite liquid scintillation cocktail (MP Biomedicals, USA). The radioactivity (cpm) of the sample was counted using a Tri-Carb 2800TR Liquid Scintillation Analyzer (PerkinElmer) and rates of oxidation calculated as described in (Lopaschuk and Barr, 1997)

^{3}H Glycolysis: Glycolytic rates were determined through the conversion of ^{3}H-glucose to $^{3}H_2O$ via enolase which converts 2-phosphoglycerate to phosphoenolpyruvate and releases H_2O as a byproduct. The cells were cultured with 1.5 mL of RPMI-low glucose media (Sigma-Aldrich Ltd, D5546, 5.5 mmol/L) doped with 0.2 µCi/mL of Glucose, D-[5-^{3}H(N): 1mCi-37MBq] (PerkinElmer) per well of a 24-well plate (1.5 mL per 1.9 cm^2 growth area). Cells were cultured in a normoxic incubator (5% CO_2 and 21% O_2) at 37°C for 8 hours. During incubation, $^{3}H_2O$ was released into the incubation media. The supernatant containing both $^{3}H_2O$ and remaining [5-^{3}H]-glucose was collected, and the $^{3}H_2O$ was separated from the ^{3}H-glucose using a Dowex 1 x 4 chloride form, 100-200 mesh (Sigma-Aldrich Ltd) anion exchange column in glass Pasteur pipettes. Preparation of the Dowex was made by adding 250 g of Dowex resin into a solution containing 1.25 M NaOH and 1.61 M boric acid. The mixture was then mixed gently, and the beads were repeatedly washed with distilled H_2O until the mixture reached pH 7.5. The exchange columns were prepared in glass Pasteur pipettes. The bases of the pipettes were blocked with glass wool and filled 2/3 with Dowex. The columns were washed through twice with distilled H_2O and allowed to drain. Scintillation vials were filled with 10 mL of scintillation fluid (Ecolite liquid scintillation cocktail, MP Biomedicals, USA), and placed underneath the columns to collect samples. 200 µL of the collected incubation media from each sample was added to the Dowex column, and incubated for 15 minutes, allowing the ^{3}H-glucose to bind to the column and $^{3}H_2O$ to be eluted into the vials. Each Dowex column was washed twice with 1 mL of distilled H_2O to wash down any residual samples. The scintillation vials were collected after the columns were fully drained, and the radioactivity (cpm) was measured using a Tri-Carb 2800TR

Liquid Scintillation Analyzer (PerkinElmer) rates of glycolysis calculated as described in (Lopaschuk and Barr, 1997).

^{14}C Glucose Oxidation: Glucose oxidation were determined through the use of a standard $^{14}CO_2$ capture technique for evaluating the oxidation and decarboxylation reactions of substrates (Barr RL, 2000; Board et al., 2017; Lopaschuk and Barr, 1997). Cells were grown in a 24 well plate and incubated in 1 mL of RPMI-low glucose media doped with 0.2 μCi/mL of Glucose, [D-[^{14}C(U): 1mCi-37MBq] (PerkinElmer). Where relevant, 400 μM OA ± 120 μM WY-14643 was added to the cocktail. The $^{14}CO_2$, produced from the glucose oxidation, was trapped on KOH-soaked filter papers covering the inside of an upside down 24-well plate that was used as a lid for the apparatus. A perforated rubber gasket that had holes corresponding to each well of the 24-well plate, was placed between the two plates which were then sealed with parafilm to make sure there was no possibility for CO_2 leakage. The plate sandwich was tightly screwed into a metal frame to create a seal. The whole apparatus was then placed inside a normoxic incubator (5% CO_2 and 21% O_2) and remained sealed for the length of the experiment (8 hours at 37°C). To trigger the release of the $^{14}CO_2$ that was trapped as bicarbonate in the media, 1 mL of 70-72% perchloric acid was injected through holes in the top plate, through the gasket and into the well after the 8-hour incubation. The new hole was instantly resealed with parafilm and the samples kept for an additional 1-2 hours to allow for complete release of dissolved $^{14}CO_2$. Filter papers containing trapped $^{14}CO_2$ were manually placed into individual scintillation vials containing 10 mL of Ecolite liquid scintillation cocktail (MP Biomedicals, USA) and analysed using the scintillation counter.

Oxygen Consumption Rate Measurements using the Clark Oxygen Electrode: The rate of mitochondrial oxygen consumption (OCR) was measured using the Clark-type oxygen electrode (Brand and Nicholls, 2011; Heather et al., 2012). Cells were rinsed with DPBS and dissociated into single cells, separated into two million cells per 15 mL falcon tube, and centrifuged (1300 rpm, 4 minutes). Supernatant was discarded, and the cell pellet was resuspended in 600 μL of respiration solution. There were two different respiration solutions used: Respiration Media (100 mM KCL, 50 mM MOPS, 1 mM EGTA, 5 mM KH_2PO_4, and 1 mg/mL BSA, prepared fresh on the day of the experiment) or the media in which the cells were routinely cultured in (eg RPMI+B27). The oxygen electrodes were embedded in the reaction chambers set to 37°C. 600 μL of respiration solution containing cells were transferred into the respiratory chambers. Respiration was then measured under baseline conditions with the addition of the energy substrate of interest (10 mM pyruvate + 5 mM malate or 400 μM oleate) and after addition of the metabolic uncoupler, carbonylcyanide-p-triuoromethoxy-phenylhydrazone (10 mM FCCP; Sigma-Aldrich Ltd).

Flow Cytometry

hiPSC-CMs were dissociated to a single-cell suspension with TrypLE Express and incubated in 100 μL of Zombie Violet (ZV; 1:100, BioLegend, 405 nm wavelength) for 20 minutes in the dark at RT. The cells were spun down, the supernatant was discarded, and the cells were fixed with 4% paraformaldehyde (20 minutes, 4°C). 10^6 hiPSC-CMs per mL of DPBS were washed twice with DPBS and incubated with blocking media (DPBS, 1% bovine serum albumin [BSA], 0.05% Triton-X) to limit aspecific staining (60 minutes, RT, in the dark). The samples were incubated with the primary antibody (Monoclonal Anti-Actinin Sarcomeric; Sigma-Aldrich Ltd), diluted 1:200 in blocking media (1 hour at RT, or overnight at 4 ◦C). The samples were washed with DPBS containing 0.5% BSA, pelleted by

centrifugation (2000 rpm, 4 minutes), and then incubated with a secondary antibody (Alexa Fluor 594; Life Technologies Ltd; donkey anti-mouse; 1/200) for 1 hour at RT, and washed three more times with DPBS containing 0.5% BSA. After the last wash, each sample was resuspended in 0.6-1 mL of DPBS and transferred to an Eppendorf tube for flow cytometry. Each run also consisted of several controls: a negative (just fixed cells), ZV control (fixed ZV-treated cells), and secondary control (single stain antibody control). For conjugated antibodies, an isotype control was run as well. The samples were run on a BD Fortessa X20 cell analyzer. 30,000 events were acquired for each sample acquired, and the data was analyzed with FlowJo Software 10.0.

Immunocytochemistry

The cells were washed with DPBS, fixed in 4% paraformaldehyde (20 minutes, 4°C), and washed with DPBS. Following fixation, the cells were then permeabilized by incubated with 0.05% Triton-X for 10 minutes at RT, washed with DPBS and incubated in blocking media (DPBS, 2% BSA) for 60 minutes at RT to limit aspecific staining. Next, the samples were incubated with primary antibody (Monoclonal Anti-Actinin Sarcomeric; Sigma-Aldrich Ltd) diluted 1:500 in blocking media (1 hour at RT, or overnight at 4ºC), washed with DPBS, and incubated with secondary antibody (Alexa Fluor 594; Life Technologies Ltd; donkey anti-mouse; 1/200) for 1 hour, in the dark at RT. The sample was washed twice DPBS. DAPI stain (diluted in DPBS) was added for 5 minutes at RT, and then the slides were rinsed twice more in DPBS. The slides were mounted with 50:50 DPBS/glycerol and stored in the dark at 4ºC until ready to be imaged. Images were taken using an Olympus Confocal Microscope.

MitoTracker

MitoTracker Red CMXRos dye (Invitrogen) diffuses passively across the mitochondrial membrane in live cells and accumulates in active mitochondria. hiPSC-CMs were incubated with RPMI or non-FBS medium with 100 nM of MitoTracker Red CMXRos dye for 40 minutes at 37°C. The cells were then washed twice with DPBS and fixed in 4% paraformaldehyde for 20 minutes at 4°C. After fixation, the cells were washed with DPBS with further staining as described above. Fixed cells were imaged using an Olympus Confocal Microscope and ImageJ was used to analyze the fluorescence intensity of MitoTracker Red CMXRos dye in the cells. The level of fluorescence of randomly selected cells was analyzed by measuring the grey value of stained areas and unstained areas (background). Fluorescence intensity was calculated by taking the average and Std Dev of 10 mean grey values of selected cells minus the mean fluorescence of background.

Scanning Electron Microscopy (SEM)

SEM images of the samples were obtained using the Zeiss EVO MA10 (10 kV) to determine the scaffold and cell morphology. Scaffolds were seeded on DD15 and cultured for 1 week before fixation with 4% paraformaldehyde for 30 minutes at RT. After fixing they were washed with deionised water, frozen and freeze-dried. Freeze-dried scaffolds (non-seeded and seeded) were sputter coated in gold prior to SEM analysis.

References

Barr RL, L.G.D. (2000). Methodology for measuring in vitro/ex vivo cardiac energy metabolism. J Pharmacol Toxicol Methods. *43*, 141–152.

Board, M., Lopez, C., van den Bos, C., Callaghan, R., Clarke, K., and Carr, C. (2017). Acetoacetate is a more efficient energy-yielding substrate for human mesenchymal stem cells than glucose and generates fewer reactive oxygen species. Int. J. Biochem. Cell Biol. *88*, 75–83.

Brand, M.D., and Nicholls, D.G. (2011). Assessing mitochondrial dysfunction in cells. Biochem. J. *437*, 575.1-575.

Breckwoldt, K., Letuffe-Brenière, D., Mannhardt, I., Schulze, T., Ulmer, B., Werner, T., Benzin, A., Klampe, B., Reinsch, M.C., Laufer, S., et al. (2017). Differentiation of cardiomyocytes and generation of human engineered heart tissue. Nat. Protoc.

Chen, Q., Bruyneel, A., Clarke, K., Carr, C., and Czernuszka, J. (2012). Collagen-Based Scaffolds for Potential Application of Heart Valve Tissue Engineering. J. Tissue Sci. Eng. *S11*, 3–8.

Chen, Q., Bruyneel, A., Carr, C., and Czernuszka, J. (2013). Bio-mechanical properties of novel bi-layer collagen-elastin scaffolds for heart valve tissue engineering. In Procedia Engineering, pp. 247–254.

Hansen, A., Eder, A., Bonstrup, M., Flato, M., Mewe, M., Schaaf, S., Aksehirlioglu, B., Schwoerer, A., Uebeler, J., and Eschenhagen, T. (2010). Development of a drug screening platform based on engineered heart tissue. Circ. Res. *107*, 35–44.

Heather, L.C., Cole, M.A., Tan, J.J., Ambrose, L.J.A., Pope, S., Abd-Jamil, A.H., Carter, E.E., Dodd, M.S., Yeoh, K.K., Schofield, C.J., et al. (2012). Metabolic adaptation to chronic hypoxia in cardiac mitochondria. Basic Res Cardiol *107*, 268–279.

Livak, K.J., and Schmittgen, T.D. (2001). Analysis of relative gene expression data using real-time quantitative PCR and the 2-ΔΔCT method. Methods.

Lopaschuk, G.D., and Barr, R.L. (1997). Measurements of fatty acid and carbohydrate metabolism in the isolated working rat heart. Mol. Cell. Biochem.

Table S1: SYBER qPCR primers

Gene	Sequence (Direction)
POLR2A	AGTCCGGATGAACTGAAGCG (FW)<br>CACGTGAAACACAGGCTTGG (RV)
TBP	TGACCCAGCATCACTGTTTCTT (FW)<br>CAAGCCCTGAGCGTAAGGTG (RV)
HPRT1	CCTGGCGTCGTGATTAGTGA (FW)<br>GGGCTACAATGTGATGGCCT (RV)
B2M	AGCAGCATCATGGAGGTTTGA (FW)<br>AGCCCTCCTAGAGCTACCTG (RV)
RYR2	CTAATGTCTGGGTGGGCTGG (FW)<br>TGCTGCGTTTGATGCTTTCA (RV)
CX43	TACCAAACAGCAGCGGAGTT (FW)<br>TGGGCACCACTCTTTTGCTT (RV)
SERCA2A	CGAACCCTTGCCACTCATCT (FW)<br>CCAGTATTGCAGGTTCCAGGT (RV)
CASQ2	ACTTTCCTCTGCTCGTTGCC (FW)<br>GCTCCTCAGCAGTTGGAAGA (RV)
MYL7	GGAGTTCAAAGAAGCCTTCAGC (FW)<br>GTCCCATTGAGCTTCTCCCC (RV)
MYH6	TCCTGCGGCCCAGATTCTTC (FW)<br>TCTTCCTTGTCATCGGGCAC (RV)
MYH7	AAGGTCAAGGCCTACAAGCG (FW)<br>CCAGGGCTGAGCAGATCAA (RV)
TNNT2	GACAGAGCGGAAAAGTGGGA (FW)<br>CTCCTTGGCCTTCTCCCTC (RV)
RUNX1	AACCTCGAAGACATCGGCAG (FW)<br>GGCTGAGGGTTAAAGGCAGT (RV)
GLUT1	TGGCATCAACGCTGTCTTCT (FW)<br>CTAGCGCGATGGTCATGAGT (RV)
GLUT4	GTTCTTTCATCTTCGCCGCC (FW)<br>CAATCACCTTCTGAGGGGCA (RV)
CS	CCAGTGCTTCCTCCACGAAT (FW)<br>GGAAACGGATGCCCTCATCA (RV)
IRS1	ACTGGACATCACAGCAGAATGA (FW)<br>ACTGAAATGGATGCATCGTACC (RV)
IRS2	GCCACCATCGTGAAAGAGTG (FW)<br>CCATCCGGGAACAAGGGAAA (RV)
PDK4	TGGTCCAAGATGCCTTTGAGT (FW)<br>GTTGCCCGCATTGCATTCTT (RV)
PPARA	GGACAAGGCCTCAGGCTATC (FW)<br>AACGAATCGCGTTGTGTGAC (RV)
PGC1A	TCTCAGTAAGGGGCTGGTTG (FW)<br>ACCAGAGCAGCACACTCGAT (RV)

Table S2: TaqMan Assays used for quantitative qPCR

**Gene**	**Gene expression assay ID**
UBC	Hs00824723L
B2M	Hs99999907
PPARa	Hs00947536
PGC1a	Hs01016719
UCP3	Hs01106052
GLUT1	Hs00892681
GLUT 4	Hs00168966
PFKM	Hs00175997
PDK4	Hs01037712
PDHA1	Hs01049345
CD36	Hs01567185
ACSL1	Hs00960561
CPT1B	Hs03046298
HADHA	Hs00426191
ACADM	Hs00936584
ECI1	Hs00157239
CS	Hs02574374
